# Tumour-induced neoneurogenesis and perineural tumour growth: a mathematical approach

**DOI:** 10.1038/srep20684

**Published:** 2016-02-10

**Authors:** Georgios Lolas, Arianna Bianchi, Konstantinos N. Syrigos

**Affiliations:** 1 Center for Information Services and High Performance Computing, Q1 Technische Universität Dresden, 01062 Dresden, Germany; 2 Department of Mathematics and Maxwell Institute for Mathematical Sciences, Heriot-Watt University, Edinburgh, Scotland, EH14 4AS, UK; 3 Oncology Unit, 3rd Department of Internal Medicine, Sotiria General Hospital, Athens School of Medicine, Athens, Greece

## Abstract

It is well-known that tumours induce the formation of a lymphatic and a blood vasculature around themselves. A similar but far less studied process occurs in relation to the nervous system and is referred to as *neoneurogenesis*. The relationship between tumour progression and the nervous system is still poorly understood and is likely to involve a multitude of factors. It is therefore relevant to study tumour-nerve interactions through mathematical modelling: this may reveal the most significant factors of the plethora of interacting elements regulating neoneurogenesis. The present work is a first attempt to model the neurobiological aspect of cancer development through a system of differential equations. The model confirms the experimental observations that a tumour is able to promote nerve formation/elongation around itself, and that high levels of nerve growth factor and axon guidance molecules are recorded in the presence of a tumour. Our results also reflect the observation that high stress levels (represented by higher norepinephrine release by sympathetic nerves) contribute to tumour development and spread, indicating a mutually beneficial relationship between tumour cells and neurons. The model predictions suggest novel therapeutic strategies, aimed at blocking the stress effects on tumour growth and dissemination.

A relationship between tumours and the nervous system has been suspected since the second century AD with the work of the Greek physician Galen^1^. Traditionally, the nervous system has not been considered to be actively involved in the process of metastasis. However, recent studies have demonstrated the presence of neurons in peritumoural regions of several human tumours, and the number of tumour-associated neurons has been correlated with metastases^2^,^3^. The relative importance of pre-existing versus newly-formed neurons to metastasis is not understood. Although pre-existing peritumoural neurons are likely to be sufficient for tumour spread, recruitment of neurons into the close proximity of a tumour may increase the propensity of tumours to metastasise. Increased nerve density and/or presence of intratumoural neurons should be regarded as an additional pathway for metastasis.

Significant progress has also been made in understanding the effects of stress- and depression-mediated release of chemicals by the nervous system on tumour cell dissemination^4^,^5^. On the one hand tumour cells produce factors that induce the formation of a neural network, and on the other the newly formed nerves release neurotransmitters that affect tumour growth and migration^6^,^7^. Following the terminology suggested by Entschladen and co-workers^8^, the formation of new nerve branches is herein called *neoneurogenesis*, in analogy to lymphangiogenesis and (blood) angiogenesis.

The present model aims to investigate how solid tumours induce peripheral nerve proliferation and how different types of nerves affect tumour growth and metastasis by releasing substances such as neurotransmitters. Also, we address the question of which role stress plays in cancer progression. This model was mainly inspired by recent works that focus predominantly on prostate cancer^2^,^7^,^9^. The study in Ayala *et al.*^2^ combines *in vitro* experiments with autopsy analysis of prostate cancer patients; Magnon and collaborators^7^ explore the effects of the nervous system on tumour progression by altering nerve structure and receptor activity in mice, after orthotopically implanting human tumour cells in the animals. Since the scope of our work does not include tumorigenesis, our model simulations start with a non-zero initial condition for primary tumour cells, reflecting the tumour cells implantation described in the study by Magnon *et al.*^7^. Our aim is to investigate the further evolution of these cells and their interactions with the pre-existing prostate-surrounding nerves. The model takes major inspiration from the work by Ayala *et al.*^2^, supporting the hypothesis of a symbiosis between nerves and tumour cells.

## Biological background

### Neurons, neurotransmitters and the Autonomic Nervous System (ANS)

Neurons (or *nerve cells*) are the core components of the nervous system. The electrical signals travelling inside a neuron are converted into signals transmitted by certain chemicals (*neurotransmitters*); these are then passed to another neuron across a *synapse*. A neurotransmitter released by a nerve binds to a receptor on another cell and, according to the receptor type, induces a certain action. The collection of all the neuronal structures that together control body functions below the level of consciousness (for instance, heart and respiratory rate, digestion and pupillary dilation) constitute the Autonomic Nervous System (ANS). The ANS is in turn made of three sub-systems; here we will focus only on two of them: the Sympathetic Nervous System (SNS, also called “fight or flight” system), which is responsible for quick response processes such as raising blood pressure or accelerating heart rate, and the Parasympathetic Nervous System (PNS, also known as “rest and digest” system), which governs slower responses such as gastrointestinal functions.

### Tumour-induced neoneurogenesis

Tumours induce innervation around themselves^3^,^10^ and, in general, high levels of innervation in tumours correlate with a poor disease outcome^2^,^7^. Tumour cells have the ability to produce substances, such as Nerve Growth Factor (NGF), that stimulate the growth and improve the survival of nerve cells^11^,^12^. NGF also promotes tumour growth^11^ and inhibits aggregation of cancer cells and thus enhances tumour invasion, although this process is currently poorly understood^13^.

Tumours also release Axon Guidance Molecules (AGMs). These molecules were originally considered only for their role in the nervous system as guidance cues for axons. The term *axon guidance* denotes the process by which neurons send out axons along a precise path in order to reach the correct targets. The tip of an axon (or *growth cone*) is equipped with receptors that can sense (gradients of) chemicals, called *guidance cues*, which “tell” them where to expand^14^. In recent years, however, it was shown that many AGMs can also influence neuronal survival and migration and likely play an important role in cancer progression^15^. There are at least three different families of AGMs (semaphorins, slits and netrins), which seem to have different roles in nervous system development and cancer progression. They are also found in many different body tissues and can regulate cell migration and apoptosis (for a review of the role of AGMs in cancers, see Chédotal *et al.*^16^).

### ANS effects on tumour progression

It was originally believed that the nervous system only indirectly affected cancer development, through perineural invasion (that is, the spread of tumours along nerve fibres^17^,^18^) and modulation of the immune function^5^. Indeed, neurotransmitters regulate the cytotoxicity of T lymphocytes and natural killer cells^19^ and induce leukocyte migration^6^,^20^; the consequent immunosuppression can favour tumour growth and progression, impairing the anti-tumour response^5^,^21^. However, it is the migratory effect of neurotransmitters that first suggested a *direct* link between nerves and tumour progression. One theory for the spread of metastases from a primary tumour to a certain organ claims that circulating cancer cells are attracted and settle in a specific region of the body due to the presence of factors such as chemokines or AGMs^16^,^22^. This assumption is in agreement with the well-known “seed and soil” hypothesis^23^. In particular, several studies have shown that neurotransmitters influence the migratory activity of cancer cells, perhaps by inducing a phenotypic change towards a more motile phenotype via intracellular signalling^24^, or simply by chemotaxis^25^. In addition, some neurotransmitters also induce tumour growth^6^. Indeed, tumour cells express many receptors, including serpentine receptors^26^ to which neurotransmitters are ligands. Neurotransmitters can induce several behavioural changes in tumour cells, mostly increasing their proliferation and/or migration (a summary of such effects can be found in Lang & Bastian^6^).

## Mathematical model

**Figure 1:**
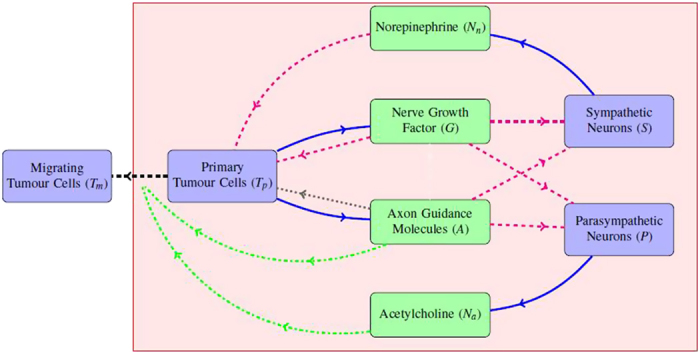
A schematic representation of the interactions among the model variables. Each variable corresponds to a rounded-corners rectangular box; note that cells are in 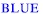 while chemicals are in 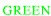. The light red-shaded rectangular area represents the main domain, that is the prostate and its immediate surroundings. Concerning the arrows, 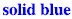 denotes production, 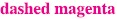 denotes enhancement of growth and/or survival (and axon extension in the case of neurons), 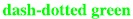 denotes migration enhancement, **dashed black** actual migration and 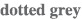 denotes apoptosis induction.

We define the *main domain* of our study as a portion of the body containing the prostate and its near surroundings, thus including both the tumour and the neighbouring nerves. All the variables, with the exception of the migrating tumour cells (see below), are average concentrations/densities over this domain, which vary in time. We develop a compartmental model in which an *extra domain* is considered for the tumour cells which leave the main domain. A schematic of the model, showing the variables and their interactions, can be found in Fig. 1.

We distinguish between *primary* tumour cells (*T*_*p*_) and *migrating* tumour cells (*T*_*m*_). The former are those that constitute the original tumour mass; when they detach and leave the orthotopic site of the tumour they are then designated migrating. The migrating cells are particularly dangerous because they have the potential to form metastases. Herein we do not explicitly account for the further development of the migrated tumour cells: our variable *T*_*m*_ represents an indication of *potential* metastasis formation.

NGF (*G*) is a neurotrophin (a kind of protein) which stimulates the growth and enhances the survival of both Sympathetic Nerve Cells (SNCs) and Parasympathetic Nerve Cells (PNCs). It has been found to be secreted by tumour cells. AGMs (*A*) also affect the survival and moreover the growth of both SNCs and PNCs. In reality, there are many kinds of AGMs, which can have completely different effects on nerve and tumour development. Here, for simplicity, we consider them as a single variable; taking into account the different types of AGMs would be one step towards improving the model in future.

The growth of both SNCs (*S*) and PNCs (*P*) is enhanced by NGF and AGMs. In addition, both types of nerve cell respond to a neurotransmitter called *acetylcholine* (*N*_*a*_), but only PNCs produce it; SNCs instead secrete *epinephrine* (also known as *adrenaline*) and *norepinephrine* (*N*_*n*_, also called *noradrenaline*). Furthermore, norepinephrine enhances tumour cell survival, growth and chemotaxis whereas acetylcholine seems to stimulate tumour cell invasion and migration^7^. Note that norepinephrine is understood to be the main tumour-related sympathetic neurotransmitter; there are less documented effects of epinephrine on tumour cell growth and dissemination^6^.

### Model equations

It is well documented that tumour cells naturally undergo mitosis (see for instance Friberg & Mattson^27^). The model accounts for this by taking constant growth rates 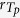 and 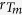 for primary and migrating tumour cells, respectively. Only a fraction of primary tumour cells exhibit proliferation; this is due to the presence of a necrotic core, that we assume to be defined by the half inner radius of the (spherical) tumour mass^28^. This assumption leads to the conclusion that only 7/8 of the tumour volume (and thus primary tumour cells) proliferate. Primary tumour cells are also exposed to the chemicals present in the domain which influence the tumour development. Since tumour growth is enhanced by NGF, we assume that the growth rate of *T*_*p*_ is increased in a saturating manner by this factor. It has been shown that a classic logistic equation is often not suitable for modelling tumour growth^29^. Here we include an *Allee effect* in the growth term to take into account the fact that tumour cell populations tend to die out at low densities. The Allee effect is an ecological term describing a correlation between the size and the per capita growth rate of a population; its inclusion in cancer modelling was already suggested by Korolev *et al.*^29^. The use of ecological concepts in cancer biology and modelling is a promising development in tumour research^30^. Here we take the *Allee threshold* (in the sense of Korolev *et al.*^29^) to be a function 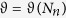 that decreases as the norepinephrine level increases. This choice reflects the observation that norepinephrine enhances tumour cell survival^31^. Tumour cells also die at a constant rate *d*_*T*_. Interestingly, some AGMs (such as netrin-1) are also thought to control tumour cell apoptosis^32^; we model this phenomenon by adding a linear dependence on *A* to the death term. Finally, another relevant aspect of tumour cell dynamics is migration. Tumour cells can spontaneously disaggregate and move away from their original site. This process is enhanced by substances produced by nerve cells and distant organs, including AGMs^33^ and acetylcholine^6^,^20^. Hence, the densities of primary and migrated tumour cells are described by the following equations:


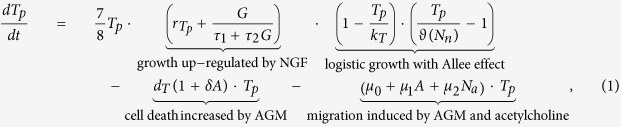



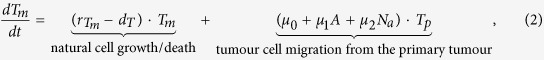


where


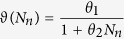


(see the supplementary material for the motivation of the definition of 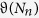).

We are interested in the effects that tumour-secreted NGF and AGMs have on the system; here the tumour secretion rate of these two growth factors is assumed to be constant^8^,^24^. We do not include other sources of NGF and AGMs in the main domain since these have a negligible effect on the dynamics that we want to study here (their effect on nerve growth in absence of tumour is implicitly included in the logistic growth of nerve cells – see below). As chemicals, both NGF and AGMs decay at constant rate *d*_*G*_ and *d*_*A*_, respectively. They are also internalised by both tumour and nerve cells, which bind them to their surface receptors. Here we assume that SNCs and PNCs bind the proteins at the same rate (namely, *γ*_2_ for NGF and *γ*_4_ for AGM). The evolution equations describing NGF and AGM dynamics in the domain are therefore given by


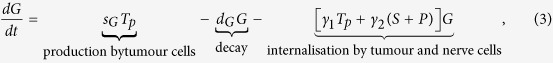



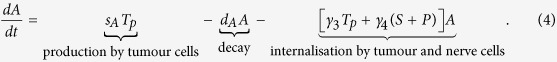


We assume that in a normal (i.e. tumour-free) setting both SNCs and PNCs grow in a logistic manner and tend to their carrying capacities *k*_*S*_ and *k*_*P*_, which are equal to their normal equilibrium values. However, when tumour cells are present nerve growth is enhanced by the secreted NGF^34^ and AGMs^35^. This additional growth is modelled by two saturating functions and is not subject to logistic limitation. This is due to the fact that, given the complex shape of neurons, it is difficult to estimate an actual maximum density. Nerve growth can also occur as axon elongation, which does not take a significant portion of space. Thus, the equations characterizing SNC and PNC rate of change are


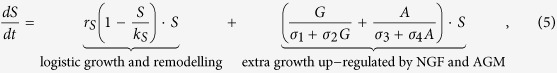



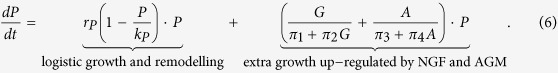


Norepinephrine and acetylcholine are produced by SNCs and PNCs, respectively^36^, at respective net rates *s*_*n*_ and *s*_*a*_ that we assume to be constant. However, these two neurotransmitters are also released by other cell types^37^,^38^ and we include constant sources *c*_*n*_, *c*_*a*_ in their equations. As chemicals, they decay at constant rates *d*_*n*_ and *d*_*a*_, respectively. Finally, they are absorbed by tumour cells^6^,^7^ at constant rates *γ*_5_ and *γ*_6_, respectively. The evolution equations for the neurotransmitters are then expressed by


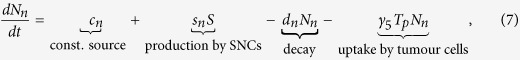



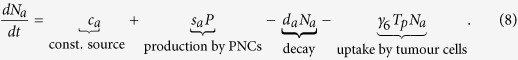


### Parameters and initial conditions

#### Parameters

Table 1 reports a list of all the parameters appearing in the model equations. Each parameter is supplied with its estimated value and units. A detailed description of the estimation of each parameter (together with used sources) can be found in the supplementary material. The parameter values were informed from the most relevant available datasets. Although these data are not “uniform” (in the sense that some are taken from experiments *in vivo* and others *in vitro*; some refer to human cell lines, others to rodents), we stress that, to the authors’ knowledge, no entirely homogeneous and complete experiment related to neoneurogenesis has been performed to date; thus a consistent estimation of the parameters is not possible. To test the robustness of the model to parameter alterations, we performed a parameter sensitivity analysis (see below).

#### Initial conditions

In order to explore model predictions in different scenarios we will run simulations under different initial conditions on the primary tumour cells. In particular, 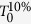 and 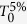 denote an initial density of primary tumour cells corresponding to 10% and 5% of the prostate volume, respectively (see the supplementary material for details). A relatively high percentage is used due to the fact that data concerning the tumour-nerve system evolution are only available for advanced stages of tumour progression (as in Ayala *et al.*^2^). We assume that a certain amount of tumour cells, corresponding to our initial data, has been implanted in previously tumour-free individuals (as done by Magnon *et al.*^7^, although there human tumour cells were implanted in mice). We also assume zero initial conditions for *T*_*m*_, NGF and AGMs, because we are interested in the growth factors produced by the tumour (see above section). All the other values are assumed to be at their normal (tumour-free) level when the model simulation starts. A list of the initial values for all the model variables can be found in Table 2.

## Results

**Figure 2:**
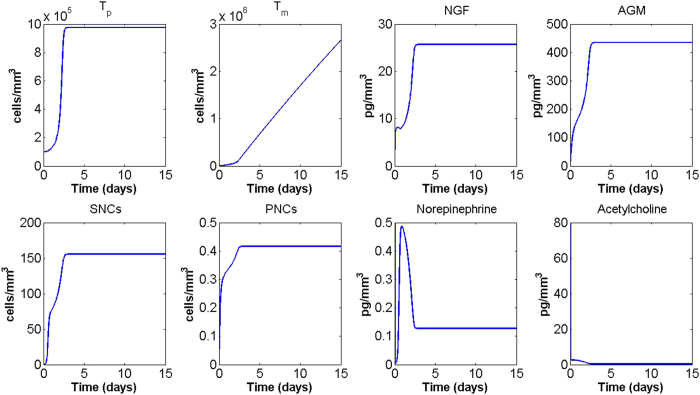
Time-course of the model variables over a period of 15 days for 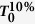.

A simulation of the system of equations (1)–(8) with initial primary tumour cell density 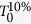 (see above) is shown in Fig. 2, where the MatLab function ode45 was used to obtain the approximate solutions. The output of the model will be compared with the experimental observations reported in Ayala *et al.*^2^ and Magnon *et al.*^7^. Note that, to the authors’ knowledge, these are the only published experiments that specifically address tumour-nerve interaction dynamics; yet, these results are not completely consistent and therefore we will not carry out a quantitative comparison. Indeed, Ayala and collaborators^2^ use three different cell lines for *in vitro* experiments (human prostate cancer, mouse neuroblastoma and rat pheochromocytoma) and data from human patients for the nerve density analysis; Magnon and co-workers implanted human prostate cancer cells into mice to collect most of their data (only the assessment of nerve density in normal tissues surrounding tumour was done on human patients). Therefore, the only possible comparison between the model results and the experimental observations is of a qualitative, rather than a quantitative, nature.

Overall, the output is in good *qualitative* agreement with the experimental observations associated with aggressive human prostate tumour as reported by Ayala and collaborators^2^. Both sympathetic and parasympathetic nerves are, in the presence of tumour, significantly increased in the region around the prostate, and the number of tumour cells leaving the domain are constantly increasing, matching the metastases-formation report in Ayala’s and Magnon’s works^2^,^7^. Concerning the primary tumour mass, our model predicts that after an initial increase it reaches a non-zero equilibrium; this is in agreement with the results of Magnon and co-workers^7^, which reports an increase in tumour mass within the prostate. Also the fact that NGF and AGM levels stay high seems realistic: NGF levels are higher in inflammation and some studies report that semaphorin 7A and netrin-1 levels are significantly elevated in patients subject to chemotherapy and some kinds of cancers, respectively. Neurotransmitters reduce rapidly to a low non-zero level following the sudden implantation of tumour cells. On the other hand, our results are not in *quantitative* agreement with Magnon *et al.*^7^; in particular, the present model reaches an equilibrium approximately 5 days after tumour cells implantation, whilst in Magnon’s report^7^ it takes weeks to observe such significant changes. This may be due to the fact that the model does not take into account other elements of the prostate environment (such as lymphatic and blood vasculature) which compete with the nervous system for growth factors and space, thus potentially slowing down the dynamics. In particular, in order to incorporate blood and lymphatic vasculature role in neoneurogenesis, one could consider extra variables representing blood and lymphatic endothelial cells as well as tumour (lymph)angiogenic growth factors. Of particular interest is the relation between NGF and vascular endothelial growth factor in prostate cancer, as proposed by Nico *et al.*^39^ and Botelho *et al.*^40^. Including the immune system also has the potential to slow down the tumour’s growth; this could be modelled for instance by considering lymphocyte dynamics or macrophage plasticity^41^. The inclusion of such extra elements is not put in practice here; however, the considered variables are sufficient to confirm the experimental evidence of tumour-nerve bilateral interactions. Also, we did investigate how perturbations in the parameter values may affect the model output and how different initial conditions will determine cancer progression.

**Figure 3:**
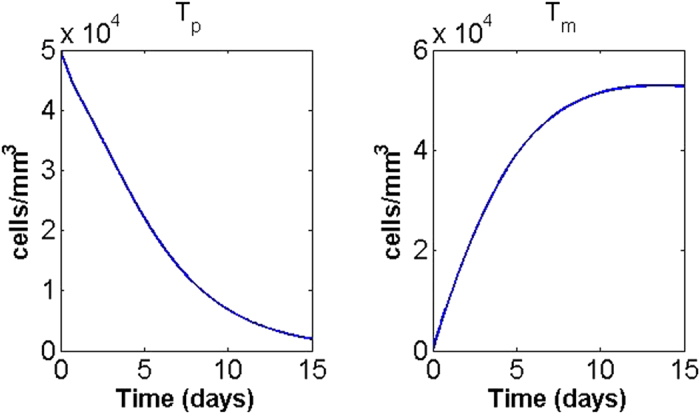
Primary and migrating tumour cells density time-course for initial condition 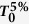.

An interesting feature of the model is that a smaller initial condition for primary tumour cells, for instance 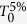, gives rise to completely different dynamics. In this case the primary tumour goes to zero after few days, while migrating tumour cells initially increase but then decrease to zero (Fig. 3). This behaviour is in accordance with the hypothesis that a tumour cell colony has to be bigger than a certain threshold in order to proliferate^29^. Note that the migrated tumour cells could cause tumour development in another site of the body where the conditions are more favourable. It is notable that the model is able to reflect this strong dependence of tumour progression on its initial conditions; this appears to be an important feature in modern cancer research inspired by ecological dynamics^29^. Our Allee threshold, lying between 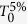 and 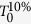, appears to be unrealistically high but, to the authors’ knowledge, no measurement of this parameter is available for comparison. In this model tumour cell survival and growth are affected only by nerves, while in reality blood vessels also contribute to tumour maintenance by providing oxygen and nutrients; this may (partially) account for the high threshold.

### Parameter sensitivity analysis

**Figure 4:**
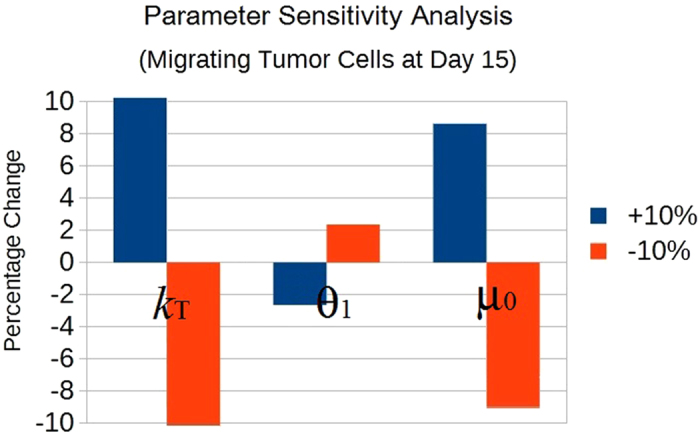
Parameter sensitivity analysis. The graph shows the effects on migrating tumour cells at day 15 after an increase (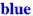) or decrease (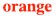) of 10% in the parameters. Here only the parameters which induced a percentage change of 2% or more are shown; they are: the tumour cell carrying capacity *k*_*T*_, the “basal” tumour cell Allee threshold *θ*_1_, and the spontaneous tumour cell migration rate *μ*_0_.

To test the robustness of the model, we performed a parameter sensitivity analysis by observing the effect that a 10% increase/reduction of each parameter value has on tumour cell densities at day 15. The model appears to be robust in the sense that final tumour cell densities are not greatly affected by perturbations in the parameter values. The only parameters that generate a change in the density of migrating tumour cells of 2% or more are reported in Fig. 4. Of these, only the tumour cell carrying capacity *k*_*T*_ has a similar effect on primary tumour cells.

### Stress and tumour progression

**Figure 5:**
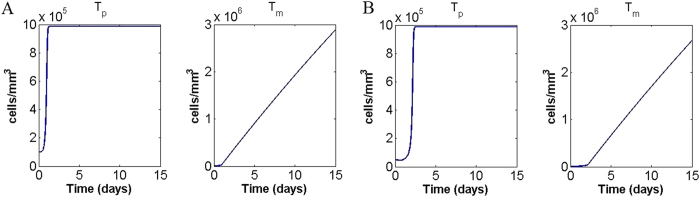
Primary and migrating tumour cells in stress conditions (simulated by multiplying *s*_*n*_ by 10) for initial conditions (**A**) 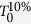 and (**B**) 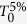 respectively.

Many cancer patients exhibit stress and depression, which are known to have an effect on the immune system and consequently tumour growth^5^,^42^. Additionally, they may have a direct effect as stress is associated with increased release of norepinephrine by the hypothalamus and sympathetic nerves^43^. Here we simulate a stress condition by increasing the norepinephrine release rate *s*_*n*_ by sympathetic nerves. Figure 5A shows the time course of primary and migrating tumour cells when *s*_*n*_ is multiplied by 10 for initial condition 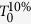. The plots show that when *s*_*n*_ is increased, the primary tumour cell density settles quickly to a higher equilibrium, while tumour cell migration is enhanced. This is in accordance with the experimental observation that stress is related to higher cancer metastasis and perhaps higher mortality^44^,^45^. Again, our results agree qualitatively (but not quantitatively) with the experimental evidence.

Another interesting prediction of our model is that for some initial conditions, such as 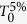, stress makes a crucial difference in tumour development. Here, if *s*_*n*_ is taken to be its baseline value, recall the primary tumour tends to zero (Fig. 3); in stress conditions (simulated by multiplying *s*_*n*_ by 10) the same initial condition leads to primary tumour growth and a constant increase of migrating tumour cells (Fig. 5B). This observation suggests that a stressful environment can affect tumour development and therapeutic efficacy, in accordance with many findings in the biological literature^5^,^31^. More experimental data are needed to precisely quantify this effect, however this already supports the potential for treatments targeting the sympathetic nervous system, as discussed by Cole & Sood^46^.

### Blocking tumour acetylcholine receptors

Regarding parasympathetic neural activity, Magnon and collaborators^7^ report that impairing the cholinergic (acetylcholine) receptors on tumour cells does not significantly affect tumour growth in the orthotopic site, but markedly reduces tumour cell spreading and metastasis. To simulate this phenomenon, we set *μ*_2_ = 0; that is, we consider tumour cells to be non-responsive to acetylcholine. In this case, we see (simulation not shown) that the number of migrated tumour cells after 15 days is reduced by about 0.5% and a similar reduction is also observed in primary tumour cell density. Thus, the model corroborates the findings of Magnon *et al.*^7^ that cholinergic receptors on tumour cells are potential clinical targets in view of limiting cancer metastasis; again, for a quantitative assessment of the potential effectiveness of this treatment one would need to include more variables in the model.

## Discussion

This work is the first mathematical confirmation of the major role played by the autonomic nervous system in promoting tumour development and progression of prostate cancer and highlights neoneurogenesis as a target for cancer drug development. In the present paper we develop a simple mathematical model for tumour neoneurogenesis and cancer progression based on recent experimental evidence; it results that, regardless of the presence of angiogenesis and lymphangiogensis, tumour-induced neoneurogenesis represents a symbiotic factor for prostate tumour. This work further expands our understanding of the process by which stress can regulate cancer aetiopathogenesis: previous research predominantly emphasised the role of the immune system in mediating stress effects on tumour growth and metastasis, while our model predicts that stress can directly affect primary tumour growth through the release of neurotransmitters. In addition, the effect of parasympathetic nerves is also captured by the model through the acetylcholine-induced tumour migration.

This model, though quite simple, gives good insights into tumour neoneurogenesis and offers many possibilities for expansion and improvement. First of all, the introduction of a spatial variable and thus the use of partial differential equations would allow a more precise description of the processes occurring during tumour neoneurogenesis. In particular, a spatial approach may be able to explain why sympathetic nerves tend to accumulate in normal tissues and only penetrate tumour edges, while parasympathetic nerves infiltrate tumour tissues^7^. Also, a more accurate description of the spatial component could allow for a distinction between axon elongation and nerve cell proliferation^2^.

The model could be further improved by considering different variables for different kinds of AGMs, which are known to have diverse effects on tumour cells^16^. In fact, circulating tumour cells are probably attracted to a specific organ by chemokines and AGMs; the fate of a new tumour cell cluster will depend on the sensitivity of the tumour cells to the specific factors and AGMs produced in the new environment.

One could also take into account the blood and lymphatic vasculatures. Guidance cues for axons also have a function in (lymph)angiogenesis^47^,^48^. Both angio-, lymphangio- and neoneuro-genesis promote metastasis formation, although in different ways; for instance, blood and lymphatic vessels offer pathways for tumour cells to disseminate, similar to perineural invasion^17^.

Another factor that could be included in the model is the immune system, which functions as a bridge between the tumour and nervous system and is the main cause of the *indirect* connections between the two (in addition, NGF also seems to be involved in immune response and inflammation^49^).

## Additional Information

**How to cite this article**: Lolas, G. *et al.* Tumour-induced neoneurogenesis and perineural tumour growth: a mathematical approach. *Sci. Rep.* **6**, 20684; doi: 10.1038/srep20684 (2016).

## Supplementary Material

Supplementary Information

## Figures and Tables

**Table 1:** A list of all the parameters appearing in the model equations.

PARAMETER	VALUE	UNITS	BIOLOGICAL INTERPRETATION
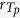	4.81 × 10^−4^	day^−1^	primary tumour cell basal growth rate
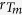	1 × 10^−4^	day^−1^	migrating tumour cell basal growth rate
*τ*_1_	134.27	pg day (mm^3^)^−1^	NGF-dependence of tumour cell growth rate
*τ*_2_	2.39	day	NGF-dependence of tumour cell growth rate
*k*_*T*_	10^6^	cells (mm^3^)^−1^	maximum tumour cell density (carrying capacity)
*θ*_1_	10^4^	cells (mm^3^)^−1^	tumour cell Allee threshold in absence of norepinephrine
*θ*_2_	1	mm^3^ pg^−1^	norepinephrine-dependence of tumour cell Allee threshold
*d*_*T*_	1.27 × 10^−2^	day^−1^	tumour cell death rate
*δ*	1.29 × 10^−2^	mm^3^ pg^−1^	AGM-dependence of tumour cell apoptosis
*μ*_0_	0.22	day^−1^	spontaneous tumour cell migration rate
*μ*_1_	9.8 × 10^−6^	mm^3^ pg^−1^ day^−1^	AGM-dependence of tumour cell migration
*μ*_2_	2 × 10^−3^	mm^3^ pg^−1^ day^−1^	acetylcholine-dependence of tumour cell migration
*s*_*G*_	2.22 × 10^−3^	pg cell^−1^ day^−1^	NGF production rate by tumour cells
*d*_*G*_	22.18	day^−1^	NGF decay rate
*γ*_1_	5.57 × 10^−5^	mm^3^ cell^−1^ day^−1^	NGF internalisation rate by tumour cells
*γ*_2_	5 × 10^−2^	mm^3^ cell^−1^ day^−1^	NGF internalisation rate by nerve cells
*s*_*A*_	5.42 × 10^−3^	pg cell^−1^ day^−1^	AGM secretion rate by tumour cells
*d*_*A*_	2.4	day^−1^	AGM decay rate
*γ*_3_	10^−5^	mm^3^ cell^−1^ day^−1^	AGM internalisation rate by tumour cells
*γ*_4_	1.47 × 10^−5^	mm^3^ cell^−1^ day^−1^	AGM internalisation rate by nerve cells
*r*_*S*_	6 × 10^−2^	day^−1^	SNC basal growth rate
*k*_*S*_	0.26	cells (mm^3^)^−1^	SNC carrying capacity
*σ*_1_	1.29 × 10^2^	pg day (mm^3^)^−1^	NGF-dependence of SNC growth rate
*σ*_2_	50	day	NGF-dependence of SNC growth rate
*σ*_3_	7.79	pg day (mm^3^)^−1^	AGM-dependence of SNC growth rate
*σ*_4_	0.01	day	AGM-dependence of SNC growth rate
*r*_*P*_	7	day^−1^	PNC basal growth rate
*k*_*P*_	0.03	cells (mm^3^)^−1^	PNC carrying capacity
*π*_1_	0.33	pg cell^−1^ day^−1^	NGF-dependence of PNC growth rate
*π*_2_	0.1	day	NGF-dependence of PNC growth rate
*π*_3_	1	pg day (mm^3^)^−1^	AGM-dependence of PNC growth rate
*π*_4_	0.01	day	AGM-dependence of PNC growth rate
*c*_*n*_	0.41	pg (mm^3^)^−1^ day^−1^	norepinephrine constant source
*s*_*n*_	1.6	pg cells^−1^ day^−1^	norepinephrine production rate by SNC
*d*_*n*_	1.66	day^−1^	norepinephrine decay rate
*γ*_5_	2 × 10^−3^	mm^3^ cell^−1^ day^−1^	norepinephrine uptake rate by tumour cells
*c*_*a*_	3.99 × 10^3^	pg (mm^3^)^−1^ day^−1^	acetylcholine constant source
*s*_*a*_	0.73	day^−1^	acetylcholine production rate by PNC
*d*_*a*_	49.91	day^−1^	acetylcholine decay rate
*γ*_6_	10^−3^	mm^3^ cell^−1^ day^−1^	acetylcholine uptake rate by tumour cells

Details can be found in the supplementary material.

**Table 2:** Values of the model variables at *t* = 0.

INIT.VALUE	VALUE	UNITS
*T*_*p*_(0)	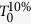 , 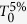	cells/mm^3^
*T*_*m*_(0)	0	cells/mm^3^
*G*(0)	0	pg/mm^3^
*A*(0)	0	pg/mm^3^
*S*(0)	0.26	cells/mm^3^
*P*(0)	0.03	cells/mm^3^
*N*_*n*_(0)	0.5	pg/mm^3^
*N*_*a*_(0)	80	pg/mm^3^

For details, see the supplementary material.
