# Supplementary material for: Tumour-induced neoneurogenesis and perineural tumour growth: a mathematical approach
Source: Sci Rep. 2016 Feb 10;6:20684. doi: 10.1038/srep20684 (PMC4748234; doi:10.1038/srep20684)
Supplement: Supplementary Information [file srep20684-s1.pdf]

# SUPPLEMENTARY INFORMATION
# Tumour-induced neoneurogenesis and perineural tumour growth: a mathematical approach

**Georgios Lolas[1,3,+,*], Arianna Bianchi[2,+], and Konstantinos N. Syrigos[3]**

[1]Center for Information Services and High Performance Computing, Q1 Technische Universität Dresden, 01062 Dresden, Germany
[2]Department of Mathematics and Maxwell Institute for Mathematical Sciences, Heriot-Watt University, Edinburgh, Scotland, EH14 4AS, UK
[3]Oncology Unit, 3rd Department of Internal Medicine, Sotiria General Hospital, Athens School of Medicine, Athens, Greece
[+]*these authors contributed equally to this work*
[*]corresponding author: glolas@med.uoa.gr

## ABSTRACT

To consolidate the discussion in the main text, we will herein expand upon the parameter estimation as well as some alternative modelling considerations addressed in the work.

The contents of the present document are reported below.

## Contents

**1 Overview** **2**
1.1 Parameters . . . 2
1.2 Initial conditions . . . 2
**2 Estimation of the model parameters** **4**
2.1 Standard sizes and weights . . . 4
2.2 Equilibrium values . . . 4
2.3 Primary tumour cells equation . . . 5
2.4 NGF equation . . . 8
2.5 AGM equation . . . 9
2.6 SNC equation . . . 10
2.7 PNC equation . . . 12
2.8 Norepinephrine (noradrenaline) equation . . . 13
2.9 Acetylcholine equation . . . 14
**References** **14**

Before starting with the parameter estimation section, we recall the full system of equations introduced in the paper.

$$\frac{dT_p}{dt} = \frac{7}{8} T_p \cdot \underbrace{\left( r_{T_p} + \frac{G}{\tau_1 + \tau_2 G} \right)}_{\text{growth upregulated by NGF}} \cdot \underbrace{\left( 1 - \frac{T_p}{k_T} \right) \cdot \left( \frac{T_p}{\vartheta(N_n)} - 1 \right)}_{\text{logistic growth with Allee effect}} - \underbrace{d_T\,(1+\delta A) \cdot T_p}_{\text{cell death induced by AGM}} - \underbrace{(\mu_0 + \mu_1 A + \mu_2 N_a) \cdot T_p}_{\text{migration induced by AGM and acetylcholine}} \quad (1)$$

$$\frac{dT_m}{dt} = \underbrace{(r_{T_m} - d_T) \cdot T_m}_{\text{natural cell growth/death}} + \underbrace{(\mu_0 + \mu_1 A + \mu_2 N_a) \cdot T_p}_{\text{tumour cell migration from the primary tumour}} \quad (2)$$

$$\frac{dG}{dt} = \underbrace{s_G T_p}_{\text{production by tumour cells}} - \underbrace{d_G G}_{\text{decay}} - \underbrace{[\gamma_1 T_p + \gamma_2 (S+P)]\, G}_{\text{internalisation by tumour and nerve cells}} \quad (3)$$

$$\frac{dA}{dt} = \underbrace{s_A T_p}_{\text{production by tumour cells}} - \underbrace{d_A A}_{\text{decay}} - \underbrace{[\gamma_3 T_p + \gamma_4 (S+P)]\, A}_{\text{internalisation by tumour and nerve cells}} \quad (4)$$

$$\frac{dS}{dt} = \underbrace{r_S \left( 1 - \frac{S}{k_S} \right) \cdot S}_{\text{logistic growth and remodelling}} + \underbrace{\left( \frac{G}{\sigma_1 + \sigma_2 G} + \frac{A}{\sigma_3 + \sigma_4 A} \right) \cdot S}_{\text{“extra growth”, upregulated by NGF and AGM}} \quad (5)$$

$$\frac{dP}{dt} = \underbrace{r_P \left( 1 - \frac{P}{k_P} \right) \cdot P}_{\text{logistic growth and remodelling}} + \underbrace{\left( \frac{G}{\pi_1 + \pi_2 G} + \frac{A}{\pi_3 + \pi_4 A} \right) \cdot P}_{\text{“extra growth”, upregulated by NGF and AGM}} \quad (6)$$

$$\frac{dN_n}{dt} = \underbrace{c_n}_{\text{const. source}} + \underbrace{s_n S}_{\text{production by SNCs}} - \underbrace{d_n N_n}_{\text{decay}} - \underbrace{\gamma_5 T_p N_n}_{\text{uptake by tumour cells}} \quad (7)$$

$$\frac{dN_a}{dt} = \underbrace{c_a}_{\text{const. source}} + \underbrace{s_a P}_{\text{production by PNCs}} - \underbrace{d_a N_a}_{\text{decay}} - \underbrace{\gamma_6 T_p N_a}_{\text{uptake by tumour cells}} \quad (8)$$

where

$$\vartheta(N_n) = \frac{\theta_1}{1 + \theta_2 N_n} \quad (9)$$

# 1 Overview

## 1.1 Parameters

Table 1 reports a list of all the parameters appearing in the model equations. Each parameter is supplied with its estimated value, units and source used (when possible) to assess it, followed by a note on the kind of dataset considered. References in brackets mean that although the parameter was not *directly* estimated from a dataset, its calculated value was inspired by the biological literature. When no data were found to inform a parameter value, this was taken to be of the same order of magnitude of another reasonably similar one. A detailed description of the estimation of each parameter can be found in Section 2.

## 1.2 Initial conditions

The initial values are listed in Table 2. As initial time $t = 0$, we take the moment at which the (primary) tumour cells occupy the (variable/adjustable) percentage $p_0$ of the prostate volume. Therefore, the initial tumour cell density is given by the expression

$$T_p(0) = \frac{p_0 \times V_{prost}}{V_{tumcell}} \times \frac{1}{V_{dom}} = p_0 \times 10^6$$

where $V_{prost}$ denotes the prostate volume, $V_{tumcell}$ the volume of a tumour cell and $V_{dom}$ the volume of the domain; their values are estimated hereunder in Section 2.1. We also start “counting” the migrating tumour cells at $t = 0$; therefore we take

| PARAMETER | VALUE | UNITS | SOURCE | NOTES |
|---|---|---|---|---|
| $r_{T_p}$ | $4.81 \times 10^{-4}$ | $\text{day}^{-1}$ | Schmid et al.[1] | human prostate cancer cells |
| $r_{T_m}$ | $1 \times 10^{-4}$ | $\text{day}^{-1}$ | estimated$\approx r_{T_p}$ | no data found |
| $\tau_1$ | 134.27 | $\text{pg day (mm}^3)^{-1}$ | Zhu et al.,[2] Sortino et al.[3] | pancreatic cancer cells/prostate adenocarcinoma cells |
| $\tau_2$ | 2.39 | day | Zhu et al.,[2] Sortino et al.[3] | pancreatic cancer cells/prostate adenocarcinoma cells |
| $k_T$ | $10^6$ | $\text{cells (mm}^3)^{-1}$ | (Park et al.[4]) | max tumour cell density $1/2 \cdot 1\text{mm}^3/V_{tumcell}$ |
| $\theta_1$ | $10^4$ | $\text{cells(mm}^3)^{-1}$ | estimated$\approx$1% of $k_T$ | no data found |
| $\theta_2$ | 1 | $\text{mm}^3\text{pg}^{-1}$ | (Chiang et al.[5]) | prostate tumour |
| $d_T$ | $1.27 \times 10^{-2}$ | $\text{day}^{-1}$ | Dachille et al.[6] | prostatic adenocarcinoma |
| $\delta$ | $1.29 \times 10^{-2}$ | $\text{mm}^3\text{pg}^{-1}$ | Castro-Rivera et al.[7] | Sema3B effect on lung and breast cancer cells (avg) |
| $\mu_0$ | 0.22 | $\text{day}^{-1}$ | Pienta et al.[8] | rat prostate tumour cells |
| $\mu_1$ | $9.8 \times 10^{-6}$ | $\text{mm}^3\text{pg}^{-1}\text{day}^{-1}$ | Herman & Meadows[9] | semaphorin-treated prostate cell line |
| $\mu_2$ | $2 \times 10^{-3}$ | $\text{mm}^3\text{pg}^{-1}\text{day}^{-1}$ | Magnon et al.[10] | carbachol-treated mice |
| $s_G$ | $2.22 \times 10^{-3}$ | $\text{pg cell}^{-1}\text{day}^{-1}$ | Dolle et al.[11] | NGF expression by breast cancer cells |
| $d_G$ | 22.18 | $\text{day}^{-1}$ | Tang et al.[12] | NGF half-life |
| $\gamma_1$ | $5.57 \times 10^{-5}$ | $\text{mm}^3\text{cell}^{-1}\text{day}^{-1}$ | Rakowicz-Szulczynska et al.[13] | internalisation of $^{125}$I-NGF by breast carcinoma cells |
| $\gamma_2$ | $5 \times 10^{-2}$ | $\text{mm}^3\text{cell}^{-1}\text{day}^{-1}$ | Claude et al.[14] | NGF internalisation by cultured rat sympathetic neurons |
| $s_A$ | $5.42 \times 10^{-3}$ | $\text{pg cell}^{-1}\text{day}^{-1}$ | Kigel et al.[15] | secreted sema3s for different tumour cell lines (avg) |
| $d_A$ | 2.4 | $\text{day}^{-1}$ | Sharova et al.[16] | semaphorins half-life |
| $\gamma_3$ | $10^{-5}$ | $\text{mm}^3\text{cell}^{-1}\text{day}^{-1}$ | estimated$\approx \gamma_4$ | no data found |
| $\gamma_4$ | $1.47 \times 10^{-5}$ | $\text{mm}^3\text{cell}^{-1}\text{day}^{-1}$ | Keino-Masu et al.[17] | binding of netrin to spinal commissural axons |
| $r_S$ | $6 \times 10^{-2}$ | $\text{day}^{-1}$ | Dolle et al.[11] | cultured sympathetic neurons from chick embryo |
| $k_S$ | 0.26 | $\text{cells (mm}^3)^{-1}$ | Magnon et al.[10] | take $k_S = S^{eq}$ (symp. neural areas in human prostate tissue) |
| $\sigma_1$ | $1.29 \times 10^2$ | $\text{pg day (mm}^3)^{-1}$ | Collins & Dawson,[18] Ruit et al.[19] | NGF-treated chicken embryo/mouse symp. neurons |
| $\sigma_2$ | 50 | day | Collins & Dawson,[18] Ruit et al.[19] | NGF-treated chicken embryo/mouse symp. neurons |
| $\sigma_3$ | 7.79 | $\text{pg day (mm}^3)^{-1}$ | Kuzirian et al.[20] | Sema4D-induced synapse formation in rodent hippocampus |
| $\sigma_4$ | 0.01 | day | (Kuzirian et al.[20]) | Sema4D-induced synapse formation in rodent hippocampus |
| $r_P$ | 7 | $\text{day}^{-1}$ | Collins & Dawson[18,21] | cultured ciliary ganglia from chicken embryo |
| $k_P$ | 0.03 | $\text{cells (mm}^3)^{-1}$ | Magnon et al.[10] | take $k_P = P^{eq}$ (parasym. neural areas in human prostate tissue) |
| $\pi_1$ | 0.33 | $\text{pg cell}^{-1}\text{day}^{-1}$ | Collins & Dawson[18] | NGF-promoted parasym. chicken embryo ciliary ganglion growth |
| $\pi_2$ | 0.1 | day | (Collins & Dawson[18]) | NGF-promoted parasym. chicken embryo ciliary ganglion growth |
| $\pi_3$ | 1 | $\text{pg day (mm}^3)^{-1}$ | estimated$\approx \sigma_3$ | no data found |
| $\pi_4$ | 0.01 | day | estimated$\approx \sigma_4$ | no data found |
| $c_n$ | 0.41 | $\text{pg (mm}^3)^{-1}\text{day}^{-1}$ | (Dodt et al.[22]) | match NE equilibrium in humans |
| $s_n$ | 1.6 | $\text{pg cells}^{-1}\text{day}^{-1}$ | Esler et al.[23] | NE release by sympathetic nerves in humans |
| $d_n$ | 1.66 | $\text{day}^{-1}$ | Taubin et al.[24] | NE half-life |
| $\gamma_5$ | $2 \times 10^{-3}$ | $\text{mm}^3\text{cell}^{-1}\text{day}^{-1}$ | Jaques et al.[25] | NE uptake by human pheochromocytoma cells |
| $c_a$ | $3.99 \times 10^3$ | $\text{pg (mm}^3)^{-1}\text{day}^{-1}$ | (Wessler et al.,[26] Watanabe et al.[27]) | match ACh equilibrium in human skin |
| $s_a$ | 0.73 | $\text{day}^{-1}$ | Paton et al.[28] | ACh output from guinea-pig ileum plexus |
| $d_a$ | 49.91 | $\text{day}^{-1}$ | Bechem et al.[29] | ACh release from guinea-pig parasym. nerve terminals |
| $\gamma_6$ | $10^{-3}$ | $\text{mm}^3\text{cell}^{-1}\text{day}^{-1}$ | estimated$\approx \gamma_5$ | no data found |

**Table 1.** A list of all the parameters appearing in the model equations (NE = norepinephrine, ACh = acetylcholine).

$T_m(0) = 0$. For $G$ and $A$, we assume their initial value to be zero, because we are interested in the NGF and AGMs produced by the tumour. All the other values are assumed to be at their normal (equilibrium) level when the model simulation starts.

| INIT.VALUE | VALUE | UNITS | SOURCE |
|---|---|---|---|
| $T_p(0)$ | $T_0^{10\%}, T_0^{5\%}$ | cells/mm$^3$ | calculated |
| $T_m(0)$ | 0 | cells/mm$^3$ | assumption |
| $G(0)$ | 0 | pg/mm$^3$ | assumption |
| $A(0)$ | 0 | pg/mm$^3$ | assumption |
| $S(0)$ | 0.26 | cells/mm$^3$ | Magnon et al.[10] |
| $P(0)$ | 0.03 | cells/mm$^3$ | Magnon et al.[10] |
| $N_n(0)$ | 0.5 | pg/mm$^3$ | Dodt et al.[22] |
| $N_a(0)$ | 80 | pg/mm$^3$ | Wessler et al.,[26] Watanabe et al.:[27] |

**Table 2.** Values of the model variables at $t = 0$.

## 2 Estimation of the model parameters

In this section, the different methods and sources used to inform the parameter values listed in Table 1 are presented in details.

### 2.1 Standard sizes and weights

#### *Domain and normal prostate sizes*

We take normal prostate size to be approximately 30mL $= 3 \times 10^4$mm$^3 = V_{prost}$.[30] Assuming a spherical shape, this implies a radius of about 20 mm.

For our model, we consider the prostate and its surroundings. Therefore we consider a slightly bigger sphere, with the same centre; say (for instance) of radius 25 mm. This leads to a domain volume $V_{dom} = 65.45 \times 10^3$mm$^3$.

#### *Tumour cell size*

In Park et al.[4] the circulating tumour cells and the cultured tumour cells in prostate cancer patients are measured; the former are found to have an average diameter of 7.97 $\mu$m, while the latter of 13.38 $\mu$m. We then take a tumour cell diameter of $10\mu$m $= 10^{-2}$mm and thus of approximate volume $V_{tumcell} = 5 \times 10^{-7}$mm$^3$ (assuming cells of spherical shape).

#### *Neurite diameter and nerve cell size*

Take neurite diameter to be about 1 $\mu$m (from Table 2.1 in Fiala & Harris[31]). Friede[32] reports that human Purkinje cell (a class of nerve cells) diameter is 27 $\mu$m. We then estimate the nerve cell volume to be approximately $10^{-5}$ mm$^3$.

#### *NGF molecular weight*

In Poduslo & Curran[33] and PhosphoSitePlus (www.phosphosite.org) NGF molecular weight is stated to be $26 \times 10^3$ Da. On the other hand, Baker[34] and Murphy et al.[35] estimated the NGF molecular weight to be between $10^4$ and $10^5$ Da. We will then assume the intermediate value $10^4$ Da $\approx 1.660 \times 10^{-8}$ pg.

#### *AGM molecular weight*

Molecular weight of Semaphorin 4D is 96,150 Da (see product at www.abcam.com). Netrin-1 molecular weight is 75 kDa = $1.245 \times 10^{-7}$ pg.[36]

#### *Norepinephrine molecular weight*

NE molecular weight = 169.17784 g/mol (from PubChem, pubchem.ncbi.nlm.nih.gov).

#### *Acetylcholine molecular weight*

Acetylcholine molecular weight = 146.20744 g/mol (from PubChem, pubchem.ncbi.nlm.nih.gov).

### 2.2 Equilibrium values

#### *Sympathetic and parasympathetic nerve density*

In Figure 7 from Magnon et al.[10] we find a quantification of sympathetic and parasympathetic (respectively) neural areas in normal human prostate tissues. From the graph, one can take a positive nerve area per field of about $1000\mu$m$^2$ for sympathetic and $100\mu$m$^2$ for parasympathetic fibres, field surface = 0.15mm$^2$. It follows that the percentage of the area occupied by nerve fibres is approximately 0.7% and 0.07 % for sympathetic and parasympathetic nerves respectively. Note that here a section is 5 $\mu$m thick. However, the staining here identify any kind of nerve fibres, and it is well known that axon size is extremely variable depending on the type (for instance, in Friede[32] it is recorded a nerve diameter of 27 $\mu$m, while Schuman et al.[37] reported a

nerve fibre layer thickness in the eye of about 100 $\mu$m). We will assume that the nerve fibres occupy the whole thickness of the sections; thus we conclude that sympathetic nerves account for 0.7% of the normal prostate tissue volume and parasympathetic ones for 0.07%.

To convert these values in an actual cells/mm$^3$ value, we recall that in 2.1 we found a domain volume of 65,450 mm$^3$. Taking the above found percentages of volume occupied by neural fibres, we have 458.1500 mm$^3$ occupied by sympathetic nerves and 45.8150 mm$^3$ by parasympathetic ones. Approximating a nerve cell a sphere of 27 $\mu$m = 27 $\times$ 10$^{-3}$ mm diameter,[32] we have that 458.1500 mm$^3$ correspond to 16,969 cells and 45.8150 mm$^3$ to 1,697 cells. Therefore, the initial sympathetic nerve density will be $S^{eq} = 16,969/65,450 \approx 0.26$ cells/mm$^3$ and the initial parasympathetic nerve density $P^{eq} = 1,697/65,450 \approx 0.03$ cells/mm$^3$.

#### *Norepinephrine level*

Dodt et al.[22] measured plasma concentration of epinephrine and norepinephrine before, during and after sleep in volunteers. They found that, although the neurotransmitters levels did not change significantly from one sleep phase to another, they increased after standing up from the horizontal position. In a final experiment, the subjects were asked to stay horizontal for 30 minutes after waking up and then stand up for and additional 30 minutes. The norepinephrine levels registered in these settings are summarised here:

- REM (rapid eye movement) and non-REM sleep: 615.4$\pm$67.8 pmol/L and 616.5$\pm$51.4 pmol/L respectively;
- after standing up: from 778.76$\pm$88.9 to 2202.7$\pm$247.55 pmol/L;
- after 30 minutes lying down plus 30 minutes standing: from 1075.2$\pm$48.9 to 3213.4$\pm$212.5 pmol/L.

So between pre- and post-sleep plasma norepinephrine levels change in a range going from 615.4 pmol/L $\approx$ 0.1 pg/mm$^3$ and 3213.4 pmol/L $\approx$ 0.5 pg/mm$^3$ (using the norepinephrine molecular weight found in 2.1). Since this value is likely to be even higher in fully awake individuals (norepinephrine is associated with stress), we will consider the latter value $N_n^{eq} = 0.5$ pg/mm$^3$.

#### *Acetylcholine level*

- Wessler et al.[26] report that "non-neuronal acetylcholine is involved in the regulation of basic cell functions" and measured acetylcholine concentration in skin biopsies from healthy volunteers. They found that "the superficial and underlying portion of skin biopsies contained 130 $\pm$ 30 and 550 $\pm$ 170 pmol/g acetylcholine, respectively".

  Since we are interested in the prostate region of the body, we will take the acetylcholine level in the "deeper" skin sample 550 pmol/g. Considering a tissue of the same density of water (1g=1mL) and the acetylcholine molecular weight reported in 2.1, we have that the acetylcholine equilibrium level is approximately $N_a^{eq} = 80$ pg/mm$^3$.
- Watanabe et al.[27] determined blood acetylcholine levels in healthy human subjects. They report that "The blood acetylcholine levels of healthy subjects varied over a wide range with a geometric mean of 0.49 $\mu$mole/liter, 90% of the levels falling into the range of 0.20 to 1.31 $\mu$mole/liter".

  Converting into our units we have $N_a^{eq} = 72$ pg/mm$^3$.

We will take $N_a^{eq} = 80$ pg/mm$^3$.

### 2.3 Primary tumour cells equation

#### *Tumour constant growth rate* $r_{T_p}$

Schmid and collaborators[1] report that prostate cancer has a very large doubling time. In particular: "Seventy-nine percent of all patients had a doubling time of more than 24 months. Twenty of 28 cancers thought to be clinically organ confined doubled at rates exceeding 48 months". We could then take $r_{T_p} = \ln 2/48\text{months} \approx 4.81 \times 10^{-4}\text{day}^{-1}$.

#### *Tumour constant death/apoptotic rate* $d_T$

Dachille et al.[6] calculate the apoptotic index (AI) of prostatic adenocarcinoma as

$$\text{AI (\%)} = 100 \times \text{apoptotic cells/total cells} .$$

The mean AI in 3,000 tumour nuclei was 1.27. We will therefore take $d_T = 1.27 \times 10^{-2}$.

To compare these *growth and death rates* with others, we see that in Stein et al.[38] it is stated that "The growth rate constants varied over a nearly 1,500-fold range, while the regression rate constants varied over a 50-fold range (Fig. 3A). Furthermore, the regression rate constants were consistently larger than the growth rate constants, with median values of $10^{-1.7}\text{day}^{-1}$ versus $10^{-2.5}\text{day}^{-1}$, respectively." These observations correspond to $r_{T_p} \approx 10^{-2.5}\text{day}^{-1}$ and $d_T \approx 10^{-1.7}\text{day}^{-1}$. Now, while $d_T$ is approximately the same computed above, $r_{T_p}$ here is bigger; this difference is explained by the fact that prostate tumour is well-known for being particularly slow in growth.

### *NGF-enhanced tumour growth* $\tau_1, \tau_2$

- Zhu et al.[2] report the dose-dependent effects of NGF on pancreatic cancer cell growth *in vitro* after 48 hours in Figure 4A. Here data are expressed as a percentage of increase or decrease of untreated controls. In particular, the data in Table 3 are recorded.

| NGF (ng/mL) | % increase of untreated controls |
|---|---|
| 6.3 | approx. 130 |
| 25 | approx. 180 |
| 100 | approx. 210 |

**Table 3.** (Recall: 1 ng/mL = 1 pg/mm$^3$.) Time = 48 hours = 2 days.

We then consider the NGF-dependent growth part in the $T_p$-equation

$$\frac{dT_p}{dt} = \left( r_{T_p} + \frac{G}{\tau_1 + \tau_2 G} - d_T \right) \cdot T_p$$

that, assuming $G$ constant, has solution

$$T(t) = T_0 \exp\left[ \left( r_{T_p} + \frac{G}{\tau_1 + \tau_2 G} - d_T \right) \cdot t \right] \qquad (10)$$

that for $G = 0$ reduces to

$$T(t) = T_0 \exp\left[ \left( r_{T_p} - d_T \right) \cdot t \right] \ , \qquad (11)$$

which will correspond to the control case.

Now, from the data in Table 3 we see that if, for example, $G = 6.3$, then

$$\frac{T_{G=6.3}(t=2)}{T_{G=0}(t=2)} = \exp\left[ \frac{G}{\tau_1 + \tau_2 G} \cdot t \right] = 1.3 \ .$$

Similarly

$$\frac{T_{G=25}(t=2)}{T_{G=0}(t=2)} = 1.8 \quad \text{and} \quad \frac{T_{G=100}(t=2)}{T_{G=0}(t=2)} = 2.1 \ .$$

We thus have a system of three equations in two unknowns $\tau_1$, $\tau_2$:

$$\begin{cases} \ln(1.3)\,[\tau_1 + 6.3 \cdot \tau_2] = 2 \cdot 6.3 \\ \ln(1.8)\,[\tau_1 + 25 \cdot \tau_2] = 2 \cdot 25 \\ \ln(2.1)\,[\tau_1 + 100 \cdot \tau_2] = 2 \cdot 100 \end{cases} \qquad (12)$$

We can then consider the following function: $y = \tau_1 + \tau_2 x$; then the system (12) corresponds to the following data points:

$$(x_1 = 6.3, y_1 = 48.02) \quad , \quad (x_2 = 25, y_2 = 85.06) \quad , \quad (x_3 = 100, y_3 = 269.56) \ .$$

Fitting the values of the parameters $\tau_1$ and $\tau_2$ to these points with MatLab functions `nlinfit` gives the following estimates: $\tau_1 = 29.54$ and $\tau_2 = 2.39$, with 95% confidence intervals $(-30.2417, 89.3126)$ and $(1.3891, 3.3943)$, respectively, given by the function `nlparci`. Note that while the estimate for $\tau_2$ seems quite accurate, the same can not be said for $\tau_1$.

- We can do a similar reasoning taking the data from Sortino et al.,[3] who investigated the effect of NGF in the androgen-dependent, prostate adenocarcinoma LNCaP cell line. The data reported by Sortino et al. are summarized in Table 4. Following a similar reasoning as the one done above with the data from Zhu et al.,[2] we find

$$\frac{T_{G=25}(t=2)}{T_{G=0}(t=2)} = \exp\left[ \frac{G}{\tau_1 + \tau_2 G} \cdot t \right] = 1.42 \quad \Rightarrow \quad \ln(1.42)\,[\tau_1 + 25 \cdot \tau_2] = 2 \cdot 25$$

and

$$\frac{T_{G=25}(t=7)}{T_{G=0}(t=7)} = \exp\left[ \frac{G}{\tau_1 + \tau_2 G} \cdot t \right] = 2.04 \quad \Rightarrow \quad \ln(2.04)\,[\tau_1 + 25 \cdot \tau_2] = 7 \cdot 25$$

respectively from the two datasets reported in Table 4 (note that 25 ng/mL = 25 pg/mm$^3$). Averaging, we obtain that $\tau_1 + 25 \cdot \tau_2 \approx 194.02$. Substituting the previously found value of $\tau_2$ ($\tau_2 = 2.39$), we get $\tau_1 = 134.27$.

We will therefore take $\tau_1 = 134.27$ and $\tau_2 = 2.39$.

| (48 hours) | cells/well ($\times 10^3$) + serum | - serum |
|---|---|---|
| Control | 153±11 | 110±2 |
| NGF (25 ng/mL) | 217±16 | 163±8 |

| (7 days) | cells/well ($\times 10^3$) |
|---|---|
| Control | 189±1.3 |
| NGF (25 ng/mL) | 385±4.6 |

**Table 4.** Data from Table 1 (left) and Table 2 (right) from Sortino et al.[3] Note that the second dataset was obtained in the presence of serum.

### *Maximum tumour cell density* $k_T$

The maximum tumour cell density is given by $1\text{mm}^3/V_{tumcell} = 2 \times 10^6$; in fact, $k_T$ corresponds to the maximum number of tumour cells that can fit in every mm$^3$. Now, because of the presence of the stroma and other cells not explicitly included in the model, we will take half of this value $k_T = 1 \times 10^6$ cells/mm$^3$.

### *Shape of* $\vartheta(N_n)$ *and values of* $\theta_1, \theta_2$

We want the function $\vartheta = \vartheta(N_n)$ to be such that $\vartheta(0) \neq 0$ (to reflect the presence of an Allee threshold in the absence of norepinephrine) and that $\vartheta$ is a decreasing function of $N_n$ (in fact, our hypothesis is that norepinephrine lowers the Allee threshold, making the tumour more likely to proliferate). We thus consider $\vartheta(N_n) = \theta_1/(1+\theta_2 N_n)$, where $\theta_1$ and $\theta_2$ are two parameters to be determined.

For $\theta_2$, we consider Figure 1 from the paper by Chiang and collaborators,[5] where the time course of prostate tumour weight is shown in control mice and in mice treated with doxazosin, an $\alpha$1-adrenergic-antagonist ($\alpha$-blocker). In the plot, we observe that in the doxazosin-treated mice the tumour weight dropped down from about 5 g to zero, while in control mice a tumour of weight around 2 g kept growing. Assuming that the doxazosin treatment blocked all the adrenergic receptors on tumour cells (thus corresponding to the case $N_n = 0$), and that in the control experiment the norepinephrine was at its equilibrium value $N_n^{eq}$, we deduce that

- when $N_n = 0$ (i.e. norepinephrine does not make any effect on tumour growth), 5 g is *below* the Allee threshold;
- when $N = N_n^{eq}$, 2 g is *above* the Allee threshold.

Now, since it is difficult to translate these tumour weights in tumour cell densities (mouse prostate size and tumour cell size are probably different from human ones), we can only use the "relative" information contained above, that is

$$\left.\begin{array}{r}\theta_1 > 5\text{ g} \\ \frac{\theta_1}{1+\theta_2 N_n^{eq}} < 2\text{ g}\end{array}\right\} \Rightarrow 1+\theta_2 N_n^{eq} > \frac{5}{2} \Rightarrow \theta_2 > 5\times 10^{-3}\,\frac{\text{mm}^3}{\text{pg}}\,.$$

We can take for instance $\theta_2 = 1$ mm$^3$/pg.

As pointed out by Korolev et al.,[39] no experiment has been done to measure the "basal" Allee threshold $\theta_1$ for any kind of tumour. We will just assume that $\theta_1$ is approximately the 1% of the carrying capacity $k_T$, i.e. $\theta_1 = 1 \times 10^4$.

### *AGM-induced tumour cell apoptosis* $\delta$

In Table 1 from Castro-Rivera and collaborators' work[7] we find a quantification of the effect of semaphorin 3B on two different kinds of cancer cells; these data are summarised in Table 5.

| Treatment | H1299 lung cancer cells | MDA-MB-231 breast cancer cells |
|---|---|---|
| Control-CM | $11 \times 10^4$ | $16 \times 10^4$ |
| SEMA3B-CM | $6 \times 10^4$ | $5 \times 10^4$ |

**Table 5.** Time = 5 days; $C_0 = 10^4$ cells/well (six-well plates)

We will then consider the following two equations for control tumour cells $T_{control}$ and for semaphorin-treated ones $T_{SEMA}$:

$$T_{control}(t) = T_0 \exp\left[\left(r_{T_p} - d_T\right)t\right] \text{ and } T_{SEMA}(t) = T_0 \exp\left[\left(r_{T_p} - d_T - \delta A\right)t\right]\,,$$

where $A$ represents the concentration of axon guidance molecule (here, semaphorin). To estimate $A$ we consider the statement "Semiquantitative assay showed an average of 15–40 ng/mL SEMA3B in the CM after transfection" in the *Materials and*

*Methods* section and the fact that the medium was diluted 1:2; in this way we approximate $A \approx 13.75$ pg/mm$^3$ (note that 1 ng/mL = 1 pg/mm$^3$). Equipped with all these values (recall: $t = 5$), we can use the data in Table 5 as follows:

$$\text{for H1299 cells: } \frac{T_{SEMA}}{T_{control}} = \exp(-\delta A t) = \frac{6}{11} \quad , \quad \text{for MDA-MB-231 cells: } \frac{T_{SEMA}}{T_{control}} = \frac{5}{16}$$

and then calculate the corresponding $\delta$ values 0.0088 and 0.0169 respectively. Taking the average, we get $\delta \approx 1.29 \times 10^{-2}$.

No data for prostate tumour cells were found to inform the value of the parameter $\delta$.

#### *Spontaneous tumour cell migration* $\mu_0$

Pienta et al.[8] observed about 1,400 colonies of (rat) prostate tumour cells after 8 days (see Figure 4 in the same reference). Without knowing how big each colony is, we will assume that 1 colony corresponds to 1 cell. Therefore, taking an exponential decay $T_p(t) = T_p(0)\exp(-\mu_0 t)$ for the tumour cells and knowing that the initial cell density was $T_p(0) = 2 \times 4 \times 10^3$ cells/mL (stated also in the work by Pienta and co-workers[8]), we can calculate $\mu_0 = 0.22$ day$^{-1}$.

#### *AGM-induced migration* $\mu_1$

In Figure 3 from Herman & Meadows' paper[9] the following % cell invasion are reported for semaphorin-treated PC-3 cells (androgen-independent prostate cell line):

$$\text{sema3A: } \sim 65\% \text{ of control} \quad , \quad \text{sema3C: } \sim 135\% \text{ of control}$$

after 20 hours incubation ($T_0 = 10^5$). The authors' comment is: "Overexpression of sema3A in PC-3 decreased the invasive characteristics of PC-3 cells by 33% compared to the untransfected cells. Sema3C, on the other hand, increased invasion by 33% compared to untransfected cells". To estimate the amount of semaphorin used in the experiment, we read: "The bacterial clones transfected with sema3A or sema3C were grown on agar plates and selected with 35 $\mu$g/mL of kanamycin". Therefore, in our equation for tumour cell migration $T_m(t) = T_0 \exp[(\mu_0 + \mu_1 A)t]$ we will take $A = 35\,\mu$g/mL $= 35 \times 10^3$ pg/mm$^3$. Finally, considering the 20-hours sema3C treatment, we have that

$$\frac{T_0 \exp[(\mu_0 + \mu_1 A)t]}{T_0 \exp(\mu_0 t)} = 1.33 \quad \Rightarrow \quad \mu_1 A t = \ln(1.33) \quad \Rightarrow \quad \mu_1 = 9.8 \times 10^{-6} \frac{\text{mm}^3}{\text{pg} \cdot \text{day}} \,.$$

#### *Acetylcholine-induced migration* $\mu_2$

Figure 3A from Magnon et al.'s paper[10] reports an *ex vivo* quantification of tumour cell invasion of pelvic lymph nodes (which drain the prostate gland). Here data are reported both for control (saline-treated) and carbachol-treated mice, and in the second case the invading tumour cells are approximately double than in the control case. Notice that since carbachol is a non-selective cholinergic (muscarinic) receptor agonist, we can consider it as a substitute of acetylcholine. Then, denoting with $c$ the carbachol amount, we can estimate from the equation $T_m(t) = T_0 \exp[(\mu_0 + \mu_2 N_a)t]$ and Figure 3A[10] that $\mu_2 c t = \ln(2)$ (since $T_0 \exp[(\mu_0 + \mu_2 c)t] \approx T_0 \exp(\mu_0 t)$). To estimate the value of $c$, we read in Magnon's paper:[10] "For experiments on the PNS, 15 days after tumour cell injection, animals received carbachol at 250 (day 0), 300 (day 1), 350 (day 2), 500 $\mu$g/kg per day (day 3) [every 12 hours, 8 divided doses]". First notice that the average of these amounts is 350 $\mu$/kg over 5 weeks, which corresponds to 10 $\mu$g/kg/day. To convert the kilos in a volume, we take water density (1 g/mL); therefore we find the approximation $c = 10$ pg/mm$^3$/day and thus $\mu_2 = 2 \times 10^{-3}$ mm$^3$pg$^{-1}$day$^{-1}$ ($t = 35$ days).

No more direct measurements of this kind of data were found by the authors.

### 2.4 NGF equation

#### *NGF decay rate* $d_G$

Tang and collaborators[12] state that "Nerve growth factor (NGF) mRNA is rapidly degraded in many non-neuronal cell types with a half-life of between 30 and 60 min". Hence, taking a half-life of 45 minutes, the resulting decay rate is $d_G = 0.0154$ min$^{-1}$ $= 22.18$ day$^{-1}$.

#### *NGF production rate by tumour cells* $s_G$

In Figure 1c from Dolle et al.[11] it is reported that after 24 hours, cultures of different lines of breast cancer cells expressed approximately 0.3 ng/(mg protein) of NGF. Considering a total protein amount of 300 pg per cell (as in HeLa cells (an immortalised cell type used in biological research, derived from cervical cancer cells taken from Henrietta Lacks), we have that 1 mg = $10^9$ pg protein corresponds to approximately $3 \times 10^6$ cells. Now, we have to consider that in 24 hours the NGF also decayed; in fact the differential equation for $G$ in this case is

$$\frac{dG}{dt} = s_G T - d_G G \quad \Rightarrow \quad G(t) = \left(G(0) - \frac{s_G}{d_G} T\right) \exp(-d_G t) + \frac{s_G}{d_G} T$$

where $T$ denotes the number of tumour cells (and $G(0) = 0$ in our case). Thus, Dolle and co-workers[11] tell us that $G(t = 1\text{day}) = 0.3 \times 10^3\text{pg}$, $T = 3 \times 10^6\text{cells}$. Substituting these numbers in the equation (and taking the value of $d_G$ estimated above), we determine $s_G = 2.22 \times 10^{-3}$ pg $\cdot$ cells$^{-1} \cdot$ day$^{-1}$.

The authors did not find any suitable dataset with prostate cancer cells.

#### *NGF internalisation rate by tumour cells* $\gamma_1$

In Table 1 from Rakowicz-Szulczynska's paper[13] it is reported the internalisation of $^{125}$I-NGF after 1 hour or 24 hours incubation of different breast carcinoma and melanoma cell lines with 10 ng/mL. For SKBr5 breast carcinoma cells, we find that 33,560 molecules/cell were internalised after 24 hours incubation. Considering a NGF molecular weight of $1.660 \times 10^{-8}$ pg and knowing that the cells were seeded at density $2 \times 10^7$ cells/10 mL $= 2 \times 10^3$ cells/mm$^3$, we can write down the equality

$$\gamma_1 \times \left(2 \times 10^3 \frac{\text{cells}}{\text{mm}^3}\right) \times \left(10 \frac{\text{pg}}{\text{mm}^3}\right) = 33.56 \times 10^3 \times 1.660 \times 10^{-8} \frac{\text{pg}}{\text{mm}^3} \times 2 \times 10^3 \text{ cells} \frac{1}{\text{day}} ,$$

which leads to $\gamma_1 = 5.57 \times 10^{-5}$ mm$^3$cells$^{-1}$day$^{-1}$.

It was not possible to find data about NGF internalisation by prostate tumour cells.

#### *NGF internalisation rate by nerve cells* $\gamma_2$

We can estimate the rate of NGF internalisation by cultured neurons using the data in Figure 1 by Claude et al.[14] The plot reports the pg of $^{125}$I-NGF binding to rat sympathetic neurons versus different amounts of free NGF. It is also stated that the neurons were incubated for 140 minutes with the NGF at a density of approximately 1,000 neurons/dish in 35-mm culture dishes.

Therefore, if we have a density of free NGF equal to $G_0$, the corresponding value on the $y$-axis of Figure 1[14] corresponds to $G(t = 140\text{min}) = G_0 \exp(-\gamma_2 St)$. Then, converting these data into our units (in particular, we considered $t$ = 140 min = 0.0972 day and $S = 1000$ neurons$/35 -$ mm dish $= 0.5$ cells/mm$^3$ from the data in Figure 1,[14] assuming a 35-mm dish of 2 mL), we can use the MatLab functions `nlinfit` and `nlparci` to get an estimate for $\gamma_2$ and its 95% confidence interval respectively. The plot of the fit is reported in Figure 1 and the output gives an estimated $\gamma_2$ value of 0.048342 with 95% confidence interval $(0.0422, 0.0545)$.

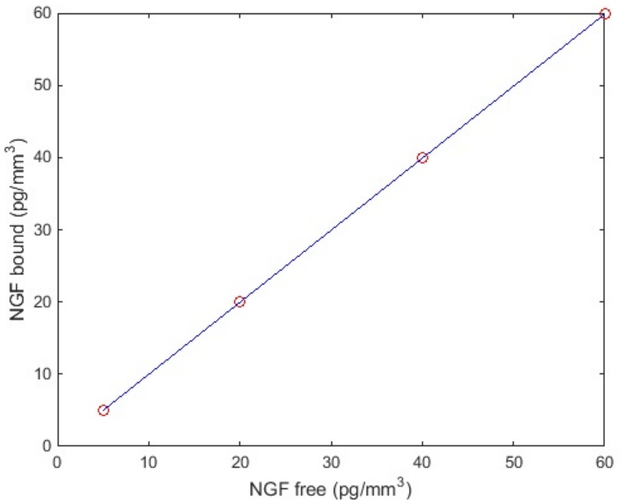


**Figure 1.** Plotting the data from Claude et al.[14] (red circles) together with the function $G(t) = G_0 \exp(-\gamma_2 St)$ (blue line) fitted to the data with the MatLab function `nlinfit`.

### 2.5 AGM equation

A large class of secreted or membrane bound axon guidance molecules are semaphorins and more specifically the so called class-3 semaphorins, that include seven family members. Class 3 semaphorins are the only secreted vertebrate semaphorins. In a recent work, Blanc et al.[40] highlighted that Semaphorin 3E is not only over-expressed in prostate cancer but also affects adhesion and motility of prostate cancer cells. They also demonstrated that all the prostate cancer cell lines that have been tested produce both the unprocessed (87kDa) and processed (61kDa) form of Sema3E. However the effect of tumour and

stromal secreted semaphorins on tumour functionalities such as migration, apoptosis, growth and invasion is likely to depend on which co-receptors are expressed. Namely, sema3E act as a chemoattractant for neurons expressing NRP1 receptors, that have been found to have a high expression on prostate tumours.

#### AGM secretion rate by tumour cells $s_A$

Kigel et al.[15] estimate the concentration of secreted sema3s in conditioned medium for specific (breast) tumour cell lines. As it can be deduced by Figure 2 from the same work[15] the relative concentrations of class-3 semaphorins secreted into the medium of tumour cell lines were 1000 and 500 sema3-expression per cell. Tumour cells were incubated for 48 hours = 2 days. Take an average of the aforementioned values, we deduce that the expression of sema3 per-cell per-day is 375. Taking the molecular weight of unprocessed sema3 to be 87kDa (as described at the beginning of this section 2.5), we estimate that the secretion rate is: $s_A = 375 \times 87000 \times 1.66 \times 10^{-12}$pg cell$^{-1}$day$^{-1}$, thus $s_A \approx 5.42 \times 10^{-5}$pg cell$^{-1}$day$^{-1}$. However, Kigel and co-workers[15] highlight that the aforementioned expressed semaphorins did not affect the proliferation rate or the survival of the different semaphorin tumour producing cells. In this regard, we expect that during tumour driven neo-neurogenesis the expressed tumour secreted sema3E are 100 or 1000 higher then the estimated value, in other words we take $s_A = 5.42 \times 10^{-3}$pg cell$^{-1}$day$^{-1}$.

We did not find prostate cancer-specific data to inform this parameter.

#### AGM decay rate $d_A$

In the Supplementary Tables 1 and 2 provided by Sharova et al.[16] we find the mRNA half-life of different kinds of semaphorins. We take an average decay rate of 0.1 $\text{h}^{-1} = 2.4$ day$^{-1}$.

Manitt et al.[36] write: "Currently, little is known about the half-life of netrin-1 protein in any context".

#### AGM internalisation by nerve cells $\gamma_4$

In Figure 4 by Keino-Masu and collaborators[17] it is studied the binding of netrin(VI•V)-Fc to DCC-expressing cells (spinal commissural axons). Here the counts per minute are reported for different concentrations of netrin. Assuming the every binding corresponds to 1 molecule, and taking the netrin molecular weight $1.245 \times 10^{-7}$ pg, we can calculate the decrease of *free netrin*, that in our system is represented by the variable $A$ and in this case is modelled by the equation $A(t) = A_0 \exp(-\gamma_4 St)$. Then, having $S = 2.5 \times 10^5$ cells/24-well = 71.43 cells/mm$^3$ (24-well $\rightarrow$ 3.5 mL) and $t = 1$ minute = $6.9444 \times 10^{-4}$ days, we can fit this as a function of $A_0$, as in Figure 4.[17] The MatLab functions `nlinfit` and `nlparci` give the $\gamma_4$ estimate $1.4673 \times 10^{-5}$ with 95% confidence interval $(0.0462 \times 10^{-4}, 0.2472 \times 10^{-4})$. The plot of this fitting is reported in Figure 2.

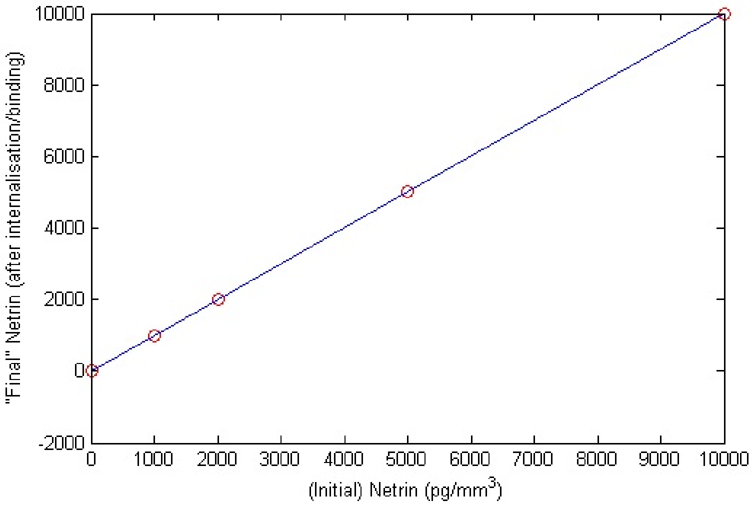


**Figure 2.** Plotting the data from Keino-Masu et al.[17] (red circles) together with the function $A(A_0) = A_0 \exp(-\gamma_4 St)$ (blue line) fitted to the data with the MatLab function `nlinfit`.

### 2.6 SNC equation

#### SNC basal growth rate $r_S$

In Table 1 presented by Dolle et al.[11] we find that 4.4% control sympathetic neurons cultured for 48 hours showed a neurite length of 29 mm. The initial cell density was $2 \times 10^3$ cells/well that, assuming a well volume of 100 mL, correspond to

$S_0 = 2\times10^{-2}$ cells/mm$^3$; moreover, taking a neurite diameter of 1 $\mu$m, we have that 29 mm neurite correspond to approximately 2.9 cells (recalling that we consider a nerve cell volume of about $10^{-5}$mm$^3$).

From Dolle et al.:[11] "Cell culture plates (96-well) were prepared by incubating each well with 100 mL of 0.1mg/mL poly-L-lysin in sterile distilled water [...] Approximately $2\times10^3$ cells, prepared from embryonic day-12 chick paravertebral sympathetic ganglia, were added to each well in 100 mL of a 1:1 mixture of [...] medium".

Hence, we conclude that after 2 days of experiment there were

$$S(t=2) = S_0 + 0.044\times S_0\times 2.9 \text{ cells} \Rightarrow S(t=2) = 2.2552\times10^{-2}\text{cells/mm}^3 = S_0\exp(2\cdot r_S)\ ,$$

from which we can calculate $r_S = 0.06$ day$^{-1}$.

### *SNC carrying capacity* $k_S$

In absence of tumour, we know that the SNC equilibrium value is $S^{eq} = 0.26$ cells/mm$^3$ (see section 2.2). We then take $k_S = S^{eq}$.

### *NGF-dependence of SNC growth rate* $\sigma_1, \sigma_2$

In Table 1 by Ruit et al.[19] the effects of NGF treatment on superior cervical ganglion cell dendritic morphology are reported; they are summarised here in Table 6.

| Treatment | Animal size | Total dendritic length ($\mu$m) |
|---|---|---|
| Control | 23.5 g | 721 |
| NGF | 23.5 g | 929 |

**Table 6.** Mouse 2.5S NGF was administered daily to mice by subcutaneous injection in a dosage of 5 mg/kg. The animals were treated for 2 weeks.

Now, if we take a dendritic diameter of 1 $\mu$m and a nerve cell volume of $10^{-5}$ mm$^3$, we have that 1 $\mu$m dendrite corresponds to about $10^{-4}$ cells. Therefore, we can "convert" the previous dendritic lengths in cells (at least roughly). For the NGF treatment, we know that it was 5 mg/kg/day for 2 weeks. If a mouse was 23.5 g, we have that each animal received $117.50\times10^6$ pg/day. Being NGF injected subcutaneously, we assume that only 1% of the dosage actually contributed to the experiment (the rest being dispersed by body fluids). Additionally, we estimate a total mouse volume of $28.57\times10^3$ mm$^3$ (knowing that mice blood volume is about 2 mL and it constitutes 7-8% of their total volume[41]) and thus we have a daily NGF supply of 41.13 pg/mm$^3$/day. Now, to calculate the effective NGF present, we have to take into account its decay. We know that NGF decay rate is $d_G = 22.18$ day$^{-1}$ (see 2.4); if we define the constant supply $s = 41.13$ pg (mm$^3$)$^{-1}$day$^{-1}$, we have that the evolution equation for $G$ in this setting is

$$\frac{dG}{dt} = s - d_G G \quad\Longrightarrow\quad G(t) = G_0 e^{d_G t} - \frac{s}{d_G}e^{-d_G t} + \frac{s}{d_G}\ .$$

Then, taking $G_0 = 0$, we have that at $t = 1$ day the amount of NGF is approximately 1.85 pg/mm$^3$. For the two-week experiment, we will then assume $G$ to be $1.85\times14 = 25.96$ pg/mm$^3$. Then, back to the $S$-equation: we recall that the part in which we are now interested is

$$\frac{dS}{dt} = \left(r_S + \frac{G}{\sigma_1+\sigma_2 G}\right)S \quad\overset{G\,\text{const}}{\Longrightarrow}\quad S(t) = S_0\exp\left[\left(r_S + \frac{G}{\sigma_1+\sigma_2 G}\right)t\right]$$

we have that at 2 weeks = 14 days

$$\frac{S_{NGF}}{S_{control}} = \exp\left(\frac{G}{\sigma_1+\sigma_2 G}\times14\right) = \frac{929}{721} = 1.29\ .$$

From this equation (recall: $G = 25.96$) we derive $\sigma_1 = 25.96\times(54.9791-\sigma_2)$. Consequently, we have that it must be $\sigma_2 < 54.9791$ in order to have $\sigma_1 > 0$.

We can derive a second equation for $\sigma_1$ and $\sigma_2$ from the experimental results reported by Collins & Dawson.[18] In fact, Table 1 from the same work[18] lists the maximal effects on neurite lengths of various additions to the culture medium. In particular, the mean total neurite length per neuron after different treatments divided by the corresponding value of the untreated control is reported. For sympathetic neurons exposed for 2 hours to 1 ng/mL NGF, the relative length is 2.47; this observation allows us to write the following equality:

$$\exp\left(\frac{G}{\sigma_1+\sigma_2 G}\times0.0833\right) = 2.47 \quad\Longrightarrow\quad \sigma_2 = 57.1782\ ,$$

the latter obtained after substituting the expression for $\sigma_1$ found previously (note that 2 hours = 0.0833 days). Notice that although $\sigma_2$ is bigger than 54.9791, the difference is small (less than one order of magnitude). This is probably due to the fact that the two references estimated $\sigma_1$, $\sigma_2$ in completely different experimental settings (for example, the experiment done by Ruit and co-workers[19] is *in vivo* while that reported by Collins & Dawson[18] is *in vitro*). Therefore it seems justified to take for instance $\sigma_2 = 50$ days and consequently $\sigma_1 \approx 129$ pg day $(\text{mm}^3)^{-1}$.

No human data were found to estimate these parameter values.

### *AGM-dependence of SNC growth rate* $\sigma_3, \sigma_4$

In Figures 1A(*ii*) and 2A Kuzirian and collaborators[20] report the synapse density after 0.5, 1, 2 and 4 hours of Sema4D treatment as % of control. In particular, it is reported that after 0.5 hours = 0.0208 days = $t_1$ of 1nM-Sema4D-treatment GABAergic synapse formation in rodent hippocampus was about 130% of control, and after 1 hour = 0.0417 days = $t_2$ it was approximately 150% of control. Now, recalling the "growth bit" of the $S$-equation

$$\frac{dS}{dt} = \left(r_S + \frac{A}{\sigma_3 + \sigma_4 A}\right) S \quad \overset{A\,\text{const}}{\Longrightarrow} \quad S(t) = S_0 \exp\left[\left(r_S + \frac{A}{\sigma_3 + \sigma_4 A}\right) t\right] ,$$

we have from the previous data points that

$$\exp\left(\frac{At_1}{\sigma_3 + \sigma_4 A}\right) = 1.3 \quad \text{and} \quad \exp\left(\frac{At_2}{\sigma_3 + \sigma_4 A}\right) = 1.5$$

(since the control corresponds to the $S(t)$ where $A = 0$). Note that taking molecular weight of 96,150 Da for $A$, we have that $A = 1$ nM $= 96.117$ pg/mm$^3$. Finally, considering the average of the two expressions above we can estimate

$$\sigma_3 + \sigma_4 A \approx \frac{1}{2}\left(\frac{At_1}{\ln(1.3)} + \frac{At_2}{\ln(1.5)}\right) \quad \Rightarrow \quad \sigma_3 \approx 8.75 - 96.12 \times \sigma_4 \, .$$

Note that we must choose $\sigma_4 < 0.0911$ in order to have $\sigma_3 > 0$. Taking for instance $\sigma_4 = 0.01$, we have consequently also $\sigma_3 = 7.79$.

No relevant data were found for human SNC.

## 2.7 PNC equation

### *PNC basal growth rate* $r_P$

In Table I Collins & Dawson[21] report that the mean total neurite length/neuron after 2¾ hours in conditioned medium was 408 $\mu$m, while in the unconditioned medium it was 118 $\mu$m (they study chicken embryo ciliary ganglia, which are parasympathetic ganglia located in the posterior orbit). Taking the latter as the initial value $P_0$, from the equation describing PNC dynamics in this context we have:

$$P(t = 2\tfrac{3}{4}\text{h} = 0.1146\text{day}) = P_0 \exp(r_P \times t)$$

$$\Rightarrow \quad 0.1146 \times r_P = \ln\left(\frac{408}{118}\right) \quad \Rightarrow \quad r_P = 10.83 \text{ day}^{-1} \, .$$

In the same reference we find another useful dataset in Table II.[21] Here it is stated that the mean elongation rate of 14 neurites (chosen to be at east 15 $\mu$m long) without any medium change was 22 $\mu$m/hour. Converting these lengths into cell numbers (using the calculations done in 2.1) and keeping in mind that 1 hour = 0.0417 days, we calculate the growth rate "per cell" $r_P$ as $22/14{\times}15 \times 1/0.0417 = 2.51$ day$^{-1}$.

Another way to determine $r_P$ could be to use the data in Table I.[18] Here the authors measure the maximal effect on ciliary (parasympathetic) and sympathetic neurite growth in various culture media after 2 hours. Considering the data regarding the "standard" conditioned medium, we have that the relative neurite length for ciliary neurons was 3.42, and for sympathetic neurons 1.81. Then, assuming an exponential growth for both cell cultures, we have that $P_0 \exp(r_P t)/S_0 \exp(r_S t) = 3.42/1.81$; furthermore, taking $P_0 = S_0$ and $t = 2\text{h} = 0.0833\text{day}$, we have that $r_P - r_S = 7.63$ day$^{-1}$. Now, recalling our previous estimate for $r_S$ ($r_S = 0.06$, see 2.6), we have $r_P = 7.70$ day$^{-1}$.

It is encouraging to see that all these three values are of the same order of magnitude. To choose an estimate for $r_P$, we take their average 7 day$^{-1}$.

The authors did not find data for human parasympathetic nerve growth.

### *PNC carrying capacity* $k_P$

In absence of tumour, we know that the PNC equilibrium value is $P^{eq} = 0.026$ cells/mm$^3$ (see section 2.2). We then take $k_P = P^{eq}$.

***NGF-dependence of PNC growth rate*** $\pi_1, \pi_2$

Collins & Dawson[18] investigated the effect of NGF on promoting the chicken embryo parasympathetic ciliary ganglion outgrowth *in vitro*. Their calculations were used to calculate the mean total length of neurites per neuron. Their calculations were based on data from neurons that had at least one neurite greater then 15 $\mu m$ in length ($\approx$ about the diameter of the neuronal soma). In this regard when they added NGF to dissociate ciliary ganglion neurons, resulted in a 2-fold increase in neurite length over untreated, control cultures.They estimated the mean total neurite length per neuron for control cultures to be $79 \pm 19\mu$m. Parasympathetic ganglion neurons were exposed to a concentration of 10ng/mL $= \frac{10\times10^3}{10^3}\,\frac{\text{pg}}{\text{mm}^3}$ per h. Just two hours after addition of NGF the ratio $\frac{P_{NGF}}{P_{control}} \approx 2.08 \pm 0.12$.

Recalling the given $P$ equation:

$$\frac{dP}{dt} = \left(r_P + \frac{G}{\pi_1 + \pi_2 G}\right) S \quad \overset{G\,\text{const}}{\Longrightarrow} \quad P(t) = P_0 \exp\left[\left(r_P + \frac{G}{\pi_1 + \pi_2 G}\right) t\right] \; ;$$

so after two hours we have

$$\frac{P_{NGF}}{P_{control}} = \exp\left(\frac{G}{\pi_1 + \pi_2 G} \times \frac{2}{24}\right) = 2.08 \; .$$

Taking into account that 2 hours $\approx \frac{2}{24}$day $= 0.083$day we deduce that

$$\frac{10}{\pi_1 + \pi_2 \times 10} \times 0.083 = \ln 2.08$$

and therefore $\pi_1 = 1.33 - 10 \times \pi_2$. Note that it must be $\pi_2 < 0.13$ in order to have $\pi_1 > 0$. We can take for example $\pi_2 = 0.1$ and thus $\pi_1 = 0.33$.

No data were found for human PNC.

## 2.8 Norepinephrine (noradrenaline) equation

***Norepinephrine production rate by SNC*** $s_n$

Regarding the norepinephrine release rate, Esler et al.[23] estimated the apparent norepinephrine release rate at rest in humans to be $0.54 \pm 0.20\mu$g/(m$^2$min) $= 777.60\,\text{pg}/\text{mm}^2{\times}\text{day}$. Note that 90% of this release rate is due to the sympathetic nerves. To convert the mm$^2$ in cells, we assume once again a nerve cell radius $r = 13.5\mu\text{m} = 13.5 \times 10^{-3}$m (see 2.1); the surface area is given by $4\pi r^2 = 4\pi(13.5 \times 10^{-3})^2 \approx 2.29 \times 10^{-3}\text{mm}^2$, thus we deduce that in 1 mm$^2$ there are $1/(2.29 \times 10^{-3}) = 436.7$ nerve cells. The norepinephrine secretion rate is then given by $s_n = 0.9 \times {}^{777.60}/_{436.7} \approx 1.6$ pg cells$^{-1}$day$^{-1}$.

***Norepinephrine decay rate*** $d_n$

Taubin et al.[24] report that the norepinephrine half-life is about 10 hours (although this value is different in different tissues). This leads to a decay rate $d_n = 1.66$ day$^{-1}$.

***Norepinephrine uptake rate by tumour cells*** $\gamma_5$

In Figure 4A by Jaques et al.[25] we find one set of measurements of NE uptake by human pheochromocytoma cells. A pheochromocytoma is a neuroendocrine tumour of the medulla of the adrenal glands; it secretes high amounts of catecholamines, mostly norepinephrine, plus epinephrine to a lesser extent. Recalling the molecular weight of NE found in 2.1 and assuming a culture volume of 1 mL (it is not better specified in the paper), we can convert the data points in Figure 4A[25] into our units and fit the function $N(t) = N_0 - N_0 \exp(-\gamma_5 T t)$ to them; note that $T$ represents the tumour cells, and that the value of this function at each time $t$ is measured as the initial substrate concentration minus the uptaken NE. Using the MatLab function `nlinfit` to fit the data we obtain an estimated $\gamma_5$ value of 0.0019926 wih 95% confidence interval $(0.0018, 0.0022)$. The plot of the fit is reported in Figure 3.

No data in this respect were found concerning prostate tumour cells.

***Norepinephrine constant source*** $c_n$

We found in 2.2 that in normal conditions (i.e. in the absence of a tumour) the level of norepinephrine is $N_n^{eq} = 0.5$ pg/mm$^3$. We can then calculate $c_n$ from the equilibrium equation

$$c_n + s_n S^{eq} - d_n N_n^{eq} = 0 \quad \Rightarrow \quad c_n \approx 0.41\,\frac{\text{pg}}{\text{mm}^3\text{day}} \; ,$$

where $S^{eq}$ and $P^{eq}$ were also found in 2.2 and $s_n$,$d_n$ were estimated above.

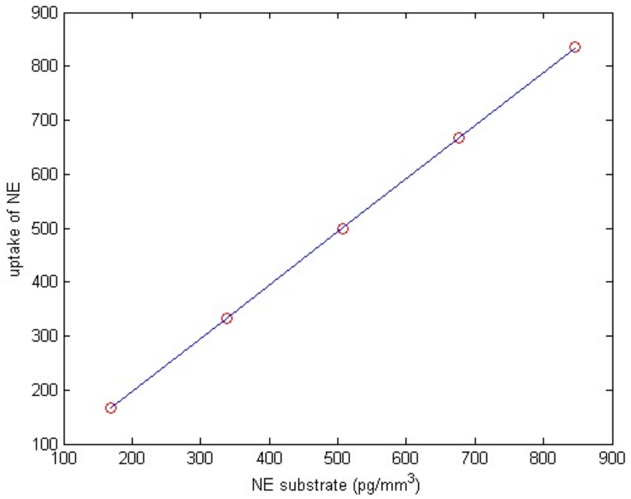


**Figure 3.** Plotting the data from Jaques et al.[25] (red circles) together with the function $N(t) = N_0 - N_0 \exp(-\gamma_5 T t)$ (blue line) fitted to the data with the MatLab function `nlinfit`.

### 2.9 Acetylcholine equation

#### *Acetylcholine production rate by PNC* $s_a$

Paton et al.[28] use the output of acetylcholine from the plexus of the guinea-pig ileum longitudinal strip to study the mechanism of acetylcholine release. The resting output is reasonably constant for a given preparation for long periods; the mean value for eighty-four experiments was about 51 ng/g·min. The evoked output, however, usually changes as stimulation is prolonged, in a manner varying with the stimulation used. Assuming a nerve cell volume of $10^{-5}$ mm$^3$ (see 2.1) and of density equal to water's one (1g/mL), we have that 1 g of parasympathetic nerves corresponds to approximately $10^8$ cells. Therefore, we estimate the acetylcholine production rate as $s_a = 0.73\,\mathrm{pg}$/cell day.

No more suitable dataset was found to inform this parameter value.

#### *Acetylcholine decay rate* $d_a$

Bechem et al.[29] studied the influence of the stimulus interval and the effect of Mn ions on facilitation of acetylcholine (ACh) release from parasympathetic nerve terminals in quiescent guinea-pig auricles (here the term *facilitation* denotes an increase in transmitter release during repetitive nerve excitation). Here we also find that when conditioning trains of stimuli were applied, a second much longer lasting component of facilitation was found ($t_{1/2} \approx 4$ s). Also, the decay to the control level displays a half time of about 20 min and can also be accelerated by frequent stimulation of the parasympathetic nerve fibres. In this regard we can estimate $d_a = 49.91\text{day}^{-1}$ (taking 20 min).

No data were found regarding acetylcholine decay rate in human tissues.

#### *Acetylcholine constant source* $c_a$

In 2.2 we estimated that in normal conditions (i.e. in the absence of a tumour) the acetylcholine level in the tissue is $N_a^{eq} = 80$ pg/mm$^3$. We can then calculate $c_a$ from the equilibrium equation

$$c_a + s_a P^{eq} - d_a N_a^{eq} = 0 \quad \Rightarrow \quad c_a \approx 3.99 \times 10^3 \,\frac{\text{pg}}{\text{mm}^3\text{day}}\,,$$

where $S^{eq}$ and $P^{eq}$ were also found in 2.2 and $s_a$,$d_a$ were estimated above.

## References


1. Schmid, H. P., McNeal, J. E. & Stamey, T. A. Observations on the doubling time of prostate cancer. The use of serial prostate-specific antigen in patients with untreated disease as a measure of increasing cancer volume. *Cancer* **71 (6),** 2031–2040 (1993).
2. Zhu, Z. W. et al. Nerve growth factor exerts differential effects on the growth of human pancreatic cancer cells. *Clin. Cancer Res.* **7 (1),** 105–112 (2001).

3. Sortino, M. A. et al. Mitogenic effect of nerve growth factor (NGF) in LNCaP prostate adenocarcinoma cells: role of the high- and low-affinity NGF receptors. *Mol. Endocrinol.* **14,** 124–136 (2000).

4. Park, S. et al. Morphological differences between circulating cells from prostate cancer patients and cultured prostate cancer cells. *PLoS ONE* **9 (1),** e85264 (2014).

5. Chiang, C. F., Son, E. L. & Wu, G. J. Oral treatment of the TRAMP mice with doxazosin suppresses prostate tumor growth and metastasis. *Prostate* **64 (4),** 408–418 (2005).

6. Dachille, G. et al. Prognostic role of cell apoptotic rate in prostate cancer: outcome of a long-time follow-up study. *Oncol. Rep.* **19 (2),** 541–545 (2008).

7. Castro-Rivera, E., Ran, S., Thorpe, P. & Minna, J. D. Semaphorin 3B (SEMA3B) induces apoptosis in lung and breast cancer, whereas VEGF165 antagonizes this effect. *Proc. Natl. Acad. Sci. U.S.A.* **101 (31),** 11432–11437 (2004).

8. Pienta, K. J. et al. Inhibition of spontaneous metastasis in a rat prostate cancer model by oral administration of modified citrus pectin. *J. Natl. Cancer Inst.* **87 (5),** 348–353 (1995).

9. Herman, J. G. & Meadows, G. G. Increased class 3 semaphorin expression modulates the invasive and adhesive properties of prostate cancer cells. *Int. J. Oncol.* **30 (5),** 1231–1238 (2007).

10. Magnon, C. et al. Autonomic nerve development contributes to prostate cancer progression. *Science* **341,** 1236361 (2013).

11. Dolle, L., El Yazidi-Belkoura, I., Adriaenssens, E., Nurcombe, V. & Hondermarck, H. Nerve growth factor overexpression and autocrine loop in breast cancer cells. *Oncogene* **22 (36),** 5592–5601 (2003).

12. Tang, B., Wang, M. & Wise, B. Nerve growth factor mRNA stability is controlled by a cis-acting instability determinant in the 3'-untranslated region. *Brain Res. Mol. Brain Res.* **46 (1-2),** 118–126 (1997).

13. Rakowicz-Szulczynska, E. M. Identification of the cell surface and nuclear receptors for NGF in a breast carcinoma cell line. *J. Cell. Physiol.* **154 (1),** 64–70 (1993).

14. Claude, P., Hawrot, E., Dunis, D. A. & Campenot, R. B. Binding, internalization, and retrograde transport of 125I-nerve growth factor in cultured rat sympathetic neurons. *J. Neurosci.* **2 (4),** 431–442 (1982).

15. Kigel, B., Varshavsky, A., Kessler, O. & Neufeld, G. Successful inhibition of tumor development by specific class-3 semaphorins is associated with expression of appropriate semaphorin receptors by tumor cells. *PLoS ONE* **3 (9)**, e3287 (2008).

16. Sharova, L. V. et al. Database for mRNA half-life of 19 977 genes obtained by DNA microarray analysis of pluripotent and differentiating mouse embryonic stem cells. *DNA Res.* **16 (1),** 45–58 (2009).

17. Keino-Masu, K. et al. Deleted in Colorectal Cancer (DCC) encodes a netrin receptor. *Cell* **87 (2),** 175–185 (1996).

18. Collins, F. & Dawson, A. An effect of nerve growth factor on parasympathetic neurite outgrowth. *Proc. Natl. Acad. Sci. U.S.A.* **80 (7)**, 2091–2094 (1983).

19. Ruit, K. G., Osborne, P. A., Schmidt, R. E., Johnson, E. M. & Snider, W. D. Nerve growth factor regulates sympathetic ganglion cell morphology and survival in the adult mouse. *J. Neurosci.* **10 (7),** 2412–2419 (1990).

20. Kuzirian, M. S., Moore, A. R., Staudenmaier, E. K., Friedel, R. H. & Paradis, S. The class 4 semaphorin Sema4D promotes the rapid assembly of GABAergic synapses in rodent hippocampus. *J. Neurosci.* **33 (21),** 8961–8973 (2013).

21. Collins, F. & Dawson, A. Conditioned medium increases the rate of neurite elongation: separation of this activity from the substratum-bound inducer of neurite outgrowth. *J. Neurosci.* **2 (8),** 1005–1010 (1982).

22. Dodt, C., Breckling, U., Derad, I., Fehm, H. L., Born, J. Plasma epinephrine and norepinephrine concentrations of healthy humans associated with nighttime sleep and morning arousal. *Hypertension* **30 (1 Pt 1),** 71–76 (1997).

23. Esler, M. et al. Determination of norepinephrine apparent release rate and clearance in humans. *Life Sciences* **25,** 1461–1470 (1979).

24. Taubin, H. L., Djahanguiri, B. & Landsberg, L. Noradrenaline concentration and turnover in different regions of the gastrointestinal tract of the rat: an approach to the evaluation of sympathetic activity in the gut.*Gut* **13 (10),** 790–795 (1972).

25. Jaques, S., Tobes, M. C. & Sisson, J. C. Sodium dependency of uptake of norepinephrine and m-iodobenzylguanidine into cultured human pheochromocytoma cells: evidence for uptake-one. *Cancer Res.* **47 (15),** 3920–3928 (1987).

26. Wessler, I., Reinheimer, T., Kilbinger, H., Bittinger, F., Kirkpatrick, C. J., Saloga, J. & Knop, J. Increased acetylcholine levels in skin biopsies of patients with atopic dermatitis. *Life Sci.* **72,** 2169–2172 (2003).

**27.** Watanabe, M., Kimura, A., Akasaka, K. & Hayashi, S. Determination of acetylcholine in human blood. *Biochem. Med. Metab. Biol.* **36,** 355–362 (1986).

**28.** Paton, W. D., Vizi, E. S. & Zar, M. A. The mechanism of acetylcholine release from parasympathetic nerves. *J. Physiol. (Lond.)* **215 (3),** 819–848 (1971).

**29.** Bechem, M., Glitsch, H. G. & Pott, L. Facilitation of acetylcholine release from cardiac parasympathetic nerve endings effect of stimulation pattern and mn ions. *Pflügers Archiv European Journal of Physiology* **391** (1981).

**30.** Nickel, J. C. Benign prostatic hyperplasia: does prostate size matter? *Rev Urol.* **5 (Suppl 4),** S12–17 (2003).

**31.** Fiala, J. C. & Harris, K. M. *Dendrites*, Ch. 1 Dendrite structure (Oxford University Press, 1999).

**32.** Friede, R. L. The relationship of body size, nerve cell size, axon length, and glial density in the cerebellum. *Proc. Natl. Acad. Sci. U.S.A.* **49,** 187–193 (1963).

**33.** Poduslo, J. F. & Curran, G. L. Permeability at the blood-brain and blood-nerve barriers of the neurotrophic factors: NGF, CNTF, NT-3, BDNF. *Brain Res. Mol. Brain Res.* **36 (2),** 280–286 (1996).

**34.** Baker, M. E. Molecular weight and structure of 7 S nerve growth factor protein. *J. Biol. Chem.* **250 (5),** 1714–1717 (1975).

**35.** Murphy, R. A., Saide, J. D., Blanchard, M. H. & Young, M. Molecular properties of the nerve growth factor secreted in mouse saliva. *Proc. Natl. Acad. Sci. U.S.A.* **74 (7),** 2672–2676 (1977).

**36.** Manitt, C. et al. Widespread expression of netrin-1 by neurons and oligodendrocytes in the adult mammalian spinal cord. *J. Neurosci.* **21 (11),** 3911–3922 (2001).

**37.** Schuman, J. S. et al. Reproducibility of nerve fiber layer thickness measurements using optical coherence tomography. *Ophthalmology* **103 (11),** 1889–1898 (1996).

**38.** Stein, W. D. et al. Tumor growth rates derived from data for patients in a clinical trial correlate strongly with patient survival: A novel strategy for evaluation of clinical trial data. *The Oncologist* **13,** 1046–1054 (2008).

**39.** Korolev, K. S., Xavier, J. B. & Gore, J. Turning ecology and evolution against cancer. *Nature Reviews Cancer* **14 (5),** 371–380 (2014).

**40.** Blanc, V. et al. A role for class 3 semaphorins in prostate cancer. *Prostate* **71 (6),** 649–658 (2011).

**41.** J. H. University, *Animal care and use committee – the mouse.*
